# Redox poise in *R. rubrum* phototrophic growth drives large-scale changes in macromolecular pathways

**DOI:** 10.1371/journal.pcbi.1013015

**Published:** 2025-06-10

**Authors:** William R. Cannon, Ethan King, Katherine A. Huening, Justin A. North

**Affiliations:** 1 Computational Mathematics Group, Pacific Northwest National Laboratory, Richland, Washington, United States of America; 2 Department of Mathematics, University of California, Riverside, California, United States of America; 3 Artificial Intelligence & Mathematical Modeling Group, Pacific Northwest National Laboratory, Richland, Washington, United States of America; 4 Department of Microbiology, The Ohio State University, Columbus, Ohio, United States of America; University of Tennessee Health Science Center College of Medicine Memphis, UNITED STATES OF AMERICA

## Abstract

During photoheterotrophic growth on organic substrates, purple nonsulfur photosynthetic bacteria like *Rhodospirillum rubrum* can acquire electrons by multiple means, including oxidation of organic substrates, oxidation of inorganic electron donors (e.g., H_2_), and by reverse electron flow from the photosynthetic electron transport chain. These electrons are stored as reduced electron-carrying cofactors (e.g., NAD(P)H and ferredoxin). The overall ratio of oxidized to reduced cofactors (e.g., NAD(P)+:NAD(P)H), or ’redox poise’, is difficult to understand or predict, as are the cellular processes for dissipating these reducing equivalents. Using physics-based models that capture mass action kinetics consistent with the thermodynamics of reactions and pathways, a range of redox conditions for heterophototrophic growth are evaluated, from conditions in which the NADP+/NADPH levels approach thermodynamic equilibrium to conditions in which the NADP+/NADPH ratio is far above the typical physiological values. Modeling predictions together with experimental measurements indicate that the redox poise of the cell results in large-scale changes in the activity of biosynthetic pathways and, thus, changes in cell macromolecule levels (DNA, RNA, proteins, and fatty acids). Furthermore, modeling predictions indicate that during phototrophic growth, reverse electron flow from the quinone pool is a minor contributor to the production of reduced cofactors (e.g., NAD(P)H) compared to other oxidative processes (H_2_ and carbon substrate oxidation). Instead, the quinone pool primarily operates to aid ATP production. The high level of ATP, in turn, drives reduction processes even when NADPH levels are relatively low compared to NADP+ by coupling ATP hydrolysis to the reductive processes. The model, in agreement with experimental measurements of macromolecule ratios of cells growing on different carbon substrates, indicates that the dynamics of nucleotide versus lipid and protein production is likely a significant mechanism of balancing oxidation and reduction in the cell.

## Author summary

During photosynthesis, plants capture light and use it to produce oxygen from water. In doing so, the energy captured from sunlight is turned into chemical energy. The question that this report seeks to answer is how certain photosynthetic bacteria dissipate this energy. We are specifically interested in the energy associated with oxidation-reduction processes (the rusting of iron is an oxidation-reduction process). In photosynthesis, light energy is initially stored as high energy electrons, ${\text{e}}^{-}$. These electrons are reduced in energy when reacted with positively charged chemical species such as protons, H^+^ to produce neutral hydrogen, ${\text{e}}^{-}+{\text{H}}^{+}\to \text{H}$. For instance in the production of reduced carbon compounds, the electrons are captured in protons, ${\text{e}}^{-}+{\text{H}}^{+}\to \text{H}$, while simultaneously carbon dioxide is reduced to a hydrogenated carbon compound, CO_2_ + 2 H → CH_2_O + 0.5 O_2_. A common example is when plants take in carbon dioxide and use it to produce sugar, C_6_H_12_O_6_. This requires that the nutrient, CO_2_ in this case, be more oxidized than the product, C_6_H_12_O_6_. More generally, the product is the cell (or biomass) itself, which typically has a molecular formula of approximately C_4_H_7_O_2_N. However, for some photosynthetic bacteria, the nutrient is already more reduced (contains more hydrogen) than the biomass. The question then is how do these bacteria dissipate the high potential electrons from photosynthesis into a reduced product if the starting material is already more reduced than the product? We find that the bacteria likely accomplish this by increasing their production of reduced biomass components such as lipids, proteins, and small acid-containing compounds.

## Introduction

Biological cells act as dissipative structures [1–5]. Dissipative structures form when the need to dissipate energy forces the material to adapt a dynamic pattern in which material movement becomes correlated, typically forming cycles, that dissipate the energy the fastest way possible. Hurricanes and tornadoes are well-known examples of dissipative structures. In tornado formation, the difference in heat between the earth’s hot surface and the cool atmosphere causes air movement (wind) to dissipate the excess energy, in accordance with the second law of thermodynamics. If the energy difference is great enough between the ground and the upper atmosphere, air movement becomes correlated and cyclical. The cyclical structure of the tornado maximizes the dissipation of energy. In thermodynamics, this is known as maximizing the entropy production. (Physical constraints may prevent the system from reaching a global maximum, however.) The cyclical structure maintains mass balance in the system, otherwise all the warm air would simply move upward and obtain heat balance but not mass balance. Consequently, the formation of the highly organized, cyclical structure of the air current is a direct consequence of maximizing the entropy production rate. While entropy is often characterized as the state of disorder of a system, this is misleading and technically incorrect. Entropy, whether thermodynamic or information entropy, is simply related to the logarithm of the probability of the system to exist in a particular state [6]. The highly organized cyclical structure of hurricanes and tornadoes is the most probable structure of the winds given the highly non-equilibrium conditions.

As defined here, dissipative structures are about dissipating energy, regardless of whether the energy is in the form of heat or material. In biological cells, the cell cycle is an example of a dissipative structure. The cell cycle of phototrophic bacteria acts to dissipate energy acquired from sunlight. Light is captured as high-potential electrons that can then be used to phosphorylate ADP to ATP via the generation of a proton-motive force. For many phototrophs, this energy is dissipated in part through fixing and reducing CO_2_ to biomass in an ATP-requiring manner. During this process, the Calvin-Benson-Bassham (CBB) cycle also acts as a dissipative cycle. Dissipative redox cycles occur throughout the cell, from the CBB cycle [7], to the tricarboxylic acid (TCA) cycle and many others (e.g., see Bar-Even *et al*. [7]). Whether flux through the TCA cycle is thermodynamically favored in the oxidative direction to produce CO_2_ or the reductive direction (Arnon-Buchanan cycle) to consume CO_2_ depends on the concentration of redox carriers in the system (i.e. NAD(P)+:NAD(P)H ratio).

**Fig 1:**
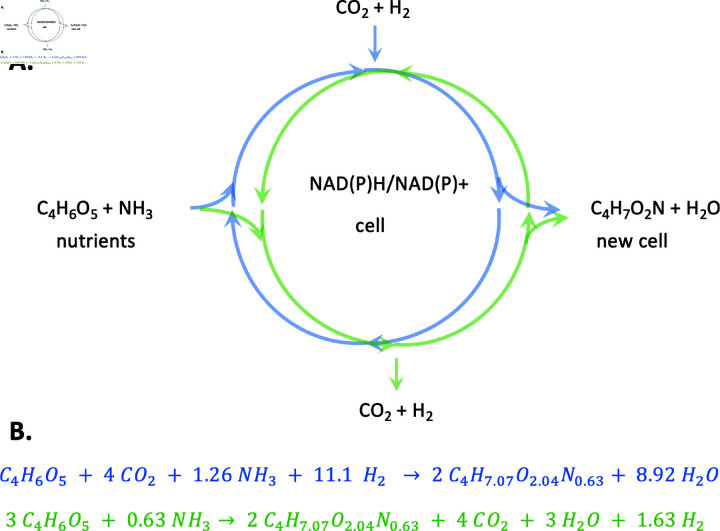
A. An abstraction of the cell acting as a dissipative structure looking at the overall chemical reaction in which the nutrients C_4_H_6_O_5_ (malate) and NH_3_ are turned into biomass, C_4_H_7_O_2_N. H_2_ simply represents an environmental reductant. The cycle can operate in either in the reductive (blue) direction and consume reductants such as H_2_ or in the oxidative direction (green) direction and produce reduced compounds. Whether net oxidation or net reduction occurs in the cell determines the *redox poise*, represented here by the cell’s ratio of NAD(P)H/NAD(P)+. In net reductive conditions, the odds of NAD(P)H/NAD(P)+ >1, while in net oxidative conditions the odds of NAD(P)H/NAD(P)+ <1. B. Examples of balanced chemical reactions for the reductive process (blue) and the oxidative process (green). Here, the biomass elemental composition, C_4_H_7.07_O_2.04_N_0.63_, is the average value found in this study.

Fig 1 is an abstract representation of energy dissipation in which the cell acts as a dissipative cycle. In this cycle, nutrients – malate (C_4_H_6_O_5_) and ammonia in this case – are taken in and biomass with a particular elemental composition is produced. Even with a compound such as malate that is relatively reduced compared to CO_2_, whether the cycle operates clockwise or counterclockwise depends on the amount of reduced compounds that are available. In Fig 1, H_2_ represents an environmental reductant that would, in principle, result in the production of reduced cofactors inside the cell. For instance, if sufficient NAD(P)H reductant can be made, then the CBB cycle or the reductive TCA cycle (Arnon-Buchanan cycle) can operate, assimilating CO_2_ and producing reduced compounds, 3-phosphoglycerate or acetyl-CoA, respectively, for synthesis of precursory metabolites and cell biomass [8]. Alternately, if sufficient reductant is not available, the idealized cell cycle of Fig 1 would operate in the counterclockwise direction, producing CO_2_ and reductant to build biomass.

For example, anoxygenic purple nonsulfur photosynthetic bacteria (PNSB) couple the dissipation of high potential electrons produced in the light harvesting complex with the synthesis of ATP through generation of a proton-motive force via bacteriochlorophyll bc_1_ complex and the Quinone (Q) cycle (cyclic photophosphorylation) [9]. Unlike in plants and algae that perform non-cyclic photophosphorylation, PNSB do not dissipate the high potential electrons through water-splitting to form NADPH. In cyclic photophosphorylation, the excited electrons return to the ground state in the light harvesting complex, and are available for another round of excitation due to photon capture [10]. However, at times reverse electron flow may occur such that NAD(P)H is formed [9,11]. The conditions under which reverse electron flow is enough to produce significant amounts NAD(P)H are not clear.

NAD(P)H reductant, whether generated from reverse electron flow, H_2_ or other electron donors, is used by PNSB to reduce carbon dioxide in the Calvin-Benson-Bassham cycle. One turn of the cycle assimilates one CO_2_ in an ATP-dependent manner,


$$D-\text{ribulose}-1,5-\text{bisphosphate}+{\text{CO}}_{2}+H_{2}\text{O}⇌....$$

⇌2 1,3-bisphospho-D-glycerate, (1)

and subsequently reduces the assimilated carbon via a hydrogenation reaction,


2 1,3-bisphospho-D-glycerate + 2 NADPH⇌

2 D-glyceraldehyde-3-phosphate + 2 NADP+. (2)

In CBB deletion strains in the PNSB *Rhodobacter sphaeroides* (*R. sph*), disposal of the NAD(P)H reducing equivalents via reduction of CO_2_ by the CBB cycle is no longer possible. As a result, the NAD(P)+ pool can become depleted [12]. This prevents photoheterotrophic growth on most organic substrates, since these are assimilated by following oxidative pathways that initially generate, not consume, NAD(P)H [13,14]. Thus, *R. sph* RubisCO deletion strains are unable to grow unless alternative NAD(P)H dissipating pathways are available (e.g. the Arnon-Buchanan reverse TCA cycle), or other adequate alternative electron acceptors are provided (e.g. DMSO and TMAO), or NAD(P)H can be dissipated via H_2_ production by nitrogenase [15,16]. Dissipation of NAD(P)H reductant when growing photoheterotrophically is essential for maintaining the redox balance and preventing inhibition of growth in all PNSB.

For other PNSB, however, it is not known how they dissipate NAD(P)H when growing photoheterotropically. During photoheterotrophic growth, the main carbon sources are short chain fatty acids and other small organic acids such as acetate, propionate, butyrate, fumarate, and malate, shown in Table 1 along with their respective carbon oxidation states. Naively, one might expect that nutrients that are more oxidized than biomass, such as fumarate, malate and acetate in Table 1, would proceed through the reductive cycle in Fig 1, while those more reduced than biomass would proceed through the oxidative cycle. However, as shown in Table 2 the overall growth reaction can operate either in the clockwise (reductive, blue) or counterclockwise (oxidative, green) direction depending on the amount of electron donors (e.g., H_2_) and thus reducing power available to the cell. Specifically, Table 2 lists several of these scenarios that can occur during growth on malate, depending on whether the cell environment is strongly reductive, mildly reductive, neutral or oxidative. In this context, oxidative refers to a net release of CO_2_, and reductive refers to a net consumption. Each scenario utilizes a different amount of reductant in the form of H_2_. The stoichiometric ratio of malate to CO_2_ used during growth under each redox condition is shown in the third column. Negative values indicate consumption while positive values indicate production. The goal of each turn of the cycle in Fig 1 is to produce biomass with the formula shown in Table 2. Likewise, Table 2 also lists similar model oxidation-reduction scenarios for growth on acetate. These scenarios represent an oversimplified model, as it assumes the cell biomass composition (CHNO ratios) are a constant for the cell across all growth substrates. However, the ability of cells to differentially produce macromolecules such as nucleotides, proteins, lipids, and carbohydrates based on the oxidation state of the growth substrate and the reducing power available to the cell must be considered, as this will in principle result in differing biomass compositions and oxidation states of the cell biomass. And yet, surprisingly, measured biomass across many species and in different environments appear to be remarkably similar [17,18].

**Table 1:** Carbon oxidation states of compounds used for photoheterotrophic growth compared to the average carbon oxidation state and average biomass formula calculated from across multiple microbes where the carbon oxidation state Z_*C*_ = (-n_*H*_ + 2n_*O*_ + 3n_*N*_)/n_*C*_ [17].

Compound	Formula	Carbon Oxidation State
Carbon dioxide	CO_2_	4
Fumarate	C_4_H_4_O_4_	1
Malate	C_4_H_6_O_5_	1
Acetate	C_2_H_4_O_2_	0
Avg Biomass	C_4_H_7.16_O_2.00_N_0.80_	-0.19
Propionate	C_3_H_5_O_2_	-0.33
Butyrate	C_4_H_7_O_2_	-0.75
Ethanol	C_2_H_6_O	-2

**Table 2:** Model redox scenarios for growth on malate (top) and acetate (bottom) with H_2_ and CO_2_ production or consumption resulting in biomass of the formula C_4_H_7.07_O_2.04_N_0.63_. Whether H_2_ and CO_2_ are produced or consumed determines whether the oxidative (green) direction or the reductive (blue) direction is taken in the dissipative cell cycles of Fig 1. In the third column, negative values indicate consumption of malate/acetate or CO_2_ and positive values indicate production. Redox Condition refers to the net oxidation or reductive environment the cell is in due to available growth substrate and electron donors (e.g. H_2_) relative to biomass carbon oxidation state.

Overall Cell				
Redox	Overall Malate Reaction			malate:CO_2_
Oxidative	3 C_4_H_6_O_5_ + 1.26 NH_3_	→	2 C_4_ H_7.07_O_2.04_N_0.63_ + 4 CO_2_ + 2.92 H_2_O + 0.90 H_2_	-3:4
~ Neutral	C_4_H_6_O_5_ + 0.63 NH_3_ + 2.63 H_2_	→	C_4_H_7.07_O_2.04_N_0.63_ + 3.04 H_2_O	-1:0
Reducing	C_4_H_6_O_5_ + 4 CO_2_ + 1.26 NH_3_ + 11.1 H_2_	→	2 C_4_ H_7.07_ O_2.04_N_0.63_ + 8.92 H_2_O	-1:-4
	Overall Acetate Reaction			acetate:CO_2_
Oxidative	3 C_2_H_4_O_2_ + 0.63 NH_3_ + 0.04 H_2_O	→	C_4_H_7.07_O_2.04_N_0.63_ + 2 CO_2_ + 3.45 H_2_	-3:2
~ Neutral	2 C_2_H_4_O_2_ + 0.63 NH_3_ + 0.55 H_2_	→	C_4_H_7.07_O_2.04_N_0.63_ + 1.96 H_2_O	-2:0
Reducing	C_2_H_4_O_2_ + 2 CO_2_ + 0.63 NH_3_ + 4.55 H_2_	→	C_4_H_7.07_O_2.04_N_0.63_ + 3.96 H_2_O	-1:-2

How PNSB dissipate reductant in central metabolism during photoheterotrophic growth on organic compounds is not obvious. During growth on malate, isotope labeling studies have been used with models to infer the direction of flux in the central metabolism of *Rhodospirillum rubrum* [19]. The models indicated that the isotope pattern was most similar to a model in which malate entered into carbon metabolism via reactions of the TCA cycle as expected. However, instead of flowing cyclically, malate proceeded through reactions for TCA metabolism in opposite redox directions, both oxidatively to oxaloacetate and reductively to succinate. Given sufficient availability of reductant, one would expect malate to proceed primarily reductively through the reductive TCA (Arnon-Buchanan) cycle. Understanding and predicting the dynamics of redox carrier concentration (i.e., redox balance) and carbon flow is a challenge for PNSB and has remained largely an open question.

A related issue is how PNSB like *R. rubrum* dissipate reductants for photoheterotrophic growth on acetate and ethanol. Acetate and ethanol are each assimilated as acetyl-CoA, some of which enters the anaplerotic ethylmalonyl-CoA pathway where molecules of acetyl-CoA are condensed and eventually reduced to malate and succinate [20]. Alternately, the anaplerotic citramalate cycle utilizes 2 molecules of acetyl-CoA, one pyruvate, and one CO_2_ in a reductive manner for the same outcome [21,22]. Malate and succinate enter the TCA cycle and are used to form biomass. Again, provided that sufficient reducing equivalents are available, one might intuitively expect that a significant percentage of the malate and succinate would proceed through the TCA cycle in the reductive direction (Arnon-Buchanan cycle).

Given this need to dissipate reductant and the metabolic versatility of PNSB, several questions arise: 1) what are the ratios of NAD(P)+:NAD(P)H and other cellular redox pairs (*i.e.,* the redox poise of the cell), 2) what reductive processes do PNSB use to dispose of reducing equivalents when they are in excess, and 3) does this change the production of cellular macromolecular components like proteins, nucleotides, carbohydrates, and lipids?

Understanding how the PNSB *Rhodospirillum rubrum* maintains electron balance by dissipating reductant and building cell mass during photoheterotrophic growth [19] is consequently important for understanding and engineering *R. rubrum* and other PNSB for biotechnology purposes, such as hydrogen, biofuels, and polyhydroxyalkonate bioplastic production. This is because these products have oxidation states as reduced or more reduced than cell biomass, potentially competing with the reduced electron-carrier cofactor pool [23,24]. The issue of reductant dissipation and biomass production is fundamentally a question of mass balance and thermodynamics.

In this report, we take advantage of new developments regarding the use of statistical thermodynamics and optimal control concepts, described in the methods section and in the literature [25–27], to model metabolism and predict how reductant is dissipated and how the dissipation modifies redox carrier ratios and biomass composition in *R. rubrum*. While Michaelis–Menten kinetics and flux-based modeling have commonly been used to study metabolism—including that of *R. rubrum* [28]—each method must be selected based on the specific scientific question asked and the assumptions underlying the model. The Michaelis–Menten equation is an approximation of a mass action rate law and does not address thermodynamics. Flux-based modeling, on the other hand, is a constraint-based approach that does not explicitly represent metabolite concentrations or their thermodynamics. Although mass action rate laws accurately incorporate kinetics and thermodynamics [29,30], as with all kinetic models, they have seen limited use because they can be challenging to develop [31,32].

The statistical thermodynamic approach used here directly determines the most likely set of rate parameters for a given steady state, thereby modeling the reaction thermodynamics [25]. However, in its current form, it does not explicitly include substrate binding to enzymes, as would a Michaelis–Menten model. It is possible to incorporate both substrate and product binding to enzymes, but doing so adds complexity that may not be necessary unless such details are critical to the research question. In addition, we employ an optimal control framework that regulates metabolite concentrations to prevent the cytosol from reaching a glassy state, which would hinder diffusion [27].

We evaluate reductant dissipation when 1) the carbon sources for growth, malate, and acetate, are less oxidized than carbon dioxide, and 2) the environment that the cell is living in is relatively oxidizing or reducing depending on the available concentration of electron donors, e.g. H_2_ in Fig 1 and NADP+/NADPH in the metabolic model. The redox conditions range from conditions in which the NADP+/NADPH levels are such that the pair are not far above thermodynamic equilibrium to redox levels in which NADP+/NADPH is far above physiological levels. All other redox pairs are coupled to NADP+/NADPH through metabolism.

Using elemental biomass composition, such as those shown in Fig 1 and Tables 2 and 3, the simulation results can be (1) directly compared to complementary experimental measurements and (2) the complex model of metabolism can be related to the abstract concept of dissipative cycles shown in Fig 1. Very few high-level cell objective functions used in modeling can be directly related to experimental observations.

**Table 3:** Primary CO_2_ assimilation reactions and enzyme catalysts. These reactions are each shown in their metabolic context of the larger model in Fig 2, where they are highlighted in orange boxes.

Enzyme/Pathway	Reaction		
RubisCO	D-ribulose-1,5-bisphosphate + CO_2_ + H_2_O	⇌	2 3-phospho-D-glycerate
EthylMal CoA	ATP + H_2_CO_3_ + propanoyl-CoA	⇌	ADP + (S)-methylmalonyl-CoA + P_*i*_
2-oxoglutarate synthase	CO_2_ + 2.0 Fedox(red) + succinyl-CoA	⇌	CoA + 2.0 Fedox(ox) + 2-oxoglutarate
isocitrate dehydrogenase	CO_2_ + NADPH + 2-oxoglutarate	⇌	NADP+ + D-threo-isocitrate
pyruvate synthase	acetyl-CoA + CO_2_ + 2.0 Fedox(red)	⇌	CoA + 2.0 Fedox(ox) + pyruvate
phosphoenolpyruvate carboxykinase	oxaloacetate + GTP	⇌	CO2 + phosphoenolpyruvate + GDP

**Fig 2:**
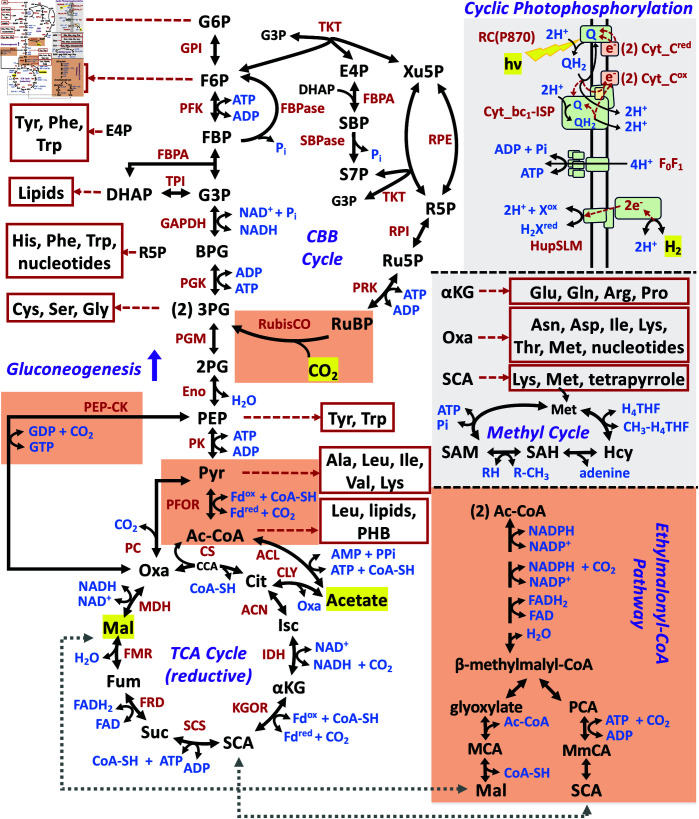
Overview of cyclic photophosphorylation by the branched-cyclic Q cycle, hydrogen uptake, and primary and secondary biosynthetic pathways of *Rhodospirillum rubrum* included in the thermo-kinetic model. Red dashed arrows indicate the flow of single electrons in the Q cycle unless otherwise noted, and each cytochrome C (Cyt_C) carries $1\;{\text{e}}^{-}$ each, so two (2) Cyt−C proteins are required. For hydrogen uptake, note the specific redox carrier that interacts with the HupSLM [Ni-Fe] hydrogenase is not known and thus indicated by X/H_2_X for a general thermodynamically acceptable redox carrier, e.g., NAD(P)+/NAD(P)H. Both the oxidative and reductive versions of the TCA cycle were tested in the modeling. The reductive TCA cycle was used in the final model. The main pathways for CO_2_ uptake or release are highlighted in orange blocks. **Metabolites** (alphabetical order): 2PG - 2-phospho-glycerate, 3PG - 3-phospho-glycerate (2 indicates the formation of 2 molecules from RuBP), Ac-CoA - acetyl-CoA, α KG - 2-keto-glutarate, BPG - 1,3-bisphospho-glycerate, Cit - citrate, CCA - citryl-CoA (intermediate of Citrate Synthase), CoA-SH - Coenzyme A, DHAP - dihydroxyacetone phosphate, E4P - erythrose-4-phosphate, F6P - fructose-6-phosphate, Fum - fumarate, G3P - glyceraldehyde-3-phosphate, G6P - glucose-6-phosphate, H4THF – Tetrahydrofolate, Hcy – homocysteine, Isc - isocitrate, LPS - lipopolysaccharide, Mal - malate, MCA - malyl-CoA, MmCA - methylmalyl-CoA, Oxa - oxaloacetate, PCA - propanoyl-CoA, PEP - phosphoenol-pyruvate, PHB - polyhydroxybutyrate, Pyr - pyruvate, R-CH3 - methylated methyl acceptor (R), Q/QH_2_ - oxidized and $2{\text{e}}^{-}$- reduced quinone, R5P - ribose-5-phosphate, Ru5P - ribulose-5-phosphate, RuBP - ribulose-1,5-bisphosphate, SAM – *S*-adenosyl-methionine, SAH – *S*-adenosyl-homocysteine, S7P - sedoheptulose-7-phosphate, SBP - sedoheptulose-1,7-bisphosphate, SCA - succinyl-CoA, Suc - succinate, Xu5P - xylulose-5-phosphate, ${\text{X}}^{\text{ox}}/{\text{H}}_{2}{\text{X}}^{\text{red}}$ - a redox carrier. All amino acids are indicated by standard 3-letter code. **Enzymes** (alphabetical order): ACL - acetate:CoA ligase (AMP-forming), ACON – aconitase, CLY – ATP-independent citrate lyase, CS - Citrate Synthase (with CCA intermediate) Cyt_bc1-ISP - complex of cytchrome b (heme b560 and b566 containing) cytochrome c1 (heme c containing) and Rieske iron sulfur protein (ISP, yellow), Cyt_C - bacterial cytochrome C2 (heme c containing), Eno – enolase, F_0_F_1_ - ATP synthasae, FBPA – fructose-1,6-bisphosphate aldolase, FBPase – fructose-1,6-bisphosphotase, FRD – fumarate reductase, FUM – fumarase, GAPDH – glyceraldehyde-3-phosphate dehydrogenase, GPI – glucose-6-phosphate isomerase, HupSLM - Uptake hydrogenase complex of small, large, and medium subunits, IDH – isocitrate dehydrogenase, KGOR – 2-keto-glutarate:ferredoxin oxidoreductase (α-KG synthase), MDH – malate dehydrogenase, NAD-ME – NAD-dependent malic enzyme, ODC – oxaloacetate decarboxylase, PEP-CK – phosphoenol-pyruvate carboxykinase, PFK – phosphofructokinase, PFOR – pyruvate-ferredoxin oxidoreductase (pyruvate synthase), PGK – 3-phosphoglycerate kinase, PGM – 3-phosphoglycerate mutase, PK – pyruvate kinase, PRK – phosphoribulokinase, RC(P870) - Type II photosynthetic reaction center with characteristic P870 pigment for photo-oxidation of ${\text{Cyt\textbackslash{}\_C}}^{\text{red}}$, RPE – ribulose-5-phosphate epimerase, RPI – ribose-5-phosphate isomerase, RubisCO – ribulose-1,5-bisphosphate oxygenase/carboxylase, SBPase – sedoheptulose-1,7-bisphosphate phosphatase, SCS – succinyl-CoA synthetase, TKT – transketolase, TPI – triose-phosphate isomerase.

The modeling results and experimental evaluation of the mass ratios of DNA, RNA, protein and fatty acids indicate that the growth substrate and resulting redox poise of the cell drives significant changes in biosynthetic pathways. Furthermore, the simulation results, combined with experimental data from the literature, imply that anaerobic photoheterotrophic growth in *R. rubrum* is actually not very reductive but lies more on the oxidative end of the range. Growth is less coupled to the production of reductant (NAD(P)H) by reverse electron flow from the quinone pool than expected. Instead, growth is mostly due to high ATP production (photophosphorylation), which drives reduction even when NAD(P)H levels are relatively low compared to NAD(P)+. Furthermore, the simulations imply that the dynamics of nucleotide versus protein production may be a significant mechanism of balancing oxidation and reduction in the cell.

## Methods

### Determining the most likely metabolism

Here, the method for formulating the mass action differential equations that describe the equations of motion of the system of coupled chemical reactions is described. Consider a reaction *j* involving *n*_*A*_ molecules of reactants *A*, and *n*_*B*_ molecules of products *B*, each with respective unsigned stoichiometric coefficients $\nu_{i,j}$ for each molecular species, and mass action rate parameters *k*_ + *j*_ and *k*_−*j*_,

$$\nu_{A,+j}{\text{n}}_{\text{A}}\underset{k_{-\text{j}}}{\overset{k_{+\text{j}}}{⇌}}\nu_{\text{B},+\text{j}}{\text{n}}_{\text{B}}.$$ (3)

The mass action rate law is,

$${\dot{\xi }}_{j}=k_{+j}n_{A}^{\nu_{A,j}}-k_{-j}n_{B}^{\nu_{B,+j}}.$$ (4)

For any mass action system, there is an infinity of steady-state solutions for Eq (4) that differ incrementally in the rate parameters *k*_ + *j*_ and *k*_−*j*_. However, given the stoichiometric matrix **S**, the infinity of solutions is also bounded such that the rate parameters and corresponding steady-state rates ${\dot{\xi }}^{ss}={\left[{\dot{\xi }}_{1},...,{\dot{\xi }}_{Z}\right]}^{T}$ must be such that the steady-state solution,

$$𝐒\cdot {\dot{\xi }}^{ss}=0,$$ (5)

is feasible. One approach to determining the most likely parameters is to carry out a search over parameter space using ensemble modeling [33,34]. Other approaches use machine learning to learn the most likely and feasible parameters [35–38]. However, the most likely mass action rate parameters *k*_ + *j*_ and *k*_−*j*_ for each of the *Z* reactions in the system can simply be derived from the maximum entropy solution to the steady state [25]. Briefly, the Boltzmann-Planck entropy S=logPr is defined as the log of the probability Pr of a system of *M* chemical species *i* with counts *n*_*i*_ and standard chemical potentials $\mu_{i}^{\circ }$. The probability of each chemical species is,

$$\theta_{i}=\frac{e^{-\mu_{i}^{\circ }/k_{B}T}}{\sum_{i}^{M}e^{-\mu_{i}^{\circ }/k_{B}T}},$$ (6)

where *T* is the temperature and *k*_*B*_ is the Boltzmann constant. The multinomial probability density function is then,

$$\text{Pr}=(\begin{array}{c} N_{tot}\\ n_{1},...,n_{M} \end{array})\prod_{i}^{M}\theta_{i}^{n_{i}},$$ (7)

where $N_{tot}=\sum_{i}n_{i}$. The maximum entropy solution is found by maximizing the chemical master equation,

$$\text{Pr}(t+\Delta t)=\text{Pr}(t)\exp\sum_{j=1}^{Z}\left(\int_{t}^{t+\Delta t}\left[{\dot{\xi }}_{j}\cdot \log K_{j}Q_{j}(t)+{\dot{\xi }}_{j}\cdot \beta \mu_{B}\sum_{i}\gamma_{i,j}\right]dt\right),$$ (8)

where ${\dot{\xi }}_{j}$ is the reaction rate, and the argument of the exponential is the entropy production rate for reaction *j*. The maximum entropy solution is found using the method of Lagrange multipliers such that the total number of reactions is finite,

$$F=\log\text{Pr}-\lambda \left(\sum_{j}\xi_{j}-c_{j}\right)$$ (9)

where λ is the undetermined multiplier and logPr=S is the Boltzmann-Planck entropy. The state of time-stationary probability has the property that the time derivative of *F* is zero such that,

$$\frac{dF}{dt}={\left[\begin{array}{l} {\dot{\xi }}_{1}^{ss}(\log\left[K_{1}Q_{1}^{-1}{\left(e^{\beta \mu_{B}}\right)}^{\sum_{i}\gamma_{i,1}}\right]-\lambda )\\ \vdots \\ {\dot{\xi }}_{Z}^{ss}(\log\left[K_{Z}Q_{Z}^{-1}{\left(e^{\beta \mu_{B}}\right)}^{\sum_{i}\gamma_{i,Z}}\right]-\lambda ) \end{array}\right]}^{T}\cdot 1={\left[\begin{array}{l} 0\\ \vdots \\ 0 \end{array}\right]}^{T}\cdot 1$$ (10)

where ${\dot{\xi }}_{j}^{ss}$ is used since this state is also a kinetic steady state and 1=1·I. In addition, the system obeys a principle of stationary action if for all reactions *j* either,

$$\log\left[K_{j}Q_{j}^{-1}e^{\beta \mu_{B}\sum_{i}\gamma_{i,j}}\right]=\lambda ,$$ (11)

or,

$${\dot{\xi }}_{j}^{ss}=0.$$ (12)

The ${\dot{\xi }}_{j}^{ss}$ can be found by solving $𝐒\;\cdot \;{\dot{\xi }}^{ss}=0$ where **S** is the stoichiometric matrix and ${\dot{\xi }}^{ss}$ is the vector of steady state reaction fluxes. The value of λ can be solved using a least squares approach.

Uncertainty in predictions due to uncertainty in the parameter space of standard free energies of reaction are provided in reference [25], as are variations in predictions due to reactions not following the maximum entropy principle. Briefly, for systems with the same steady state and only varying in rate parameters, steady state rates are the same, but concentrations of the metabolites will differ such that not every reaction has the same free energy change. For systems that vary in rate parameters but do not have the same steady-state rate, all reactions have net fluxes in the same direction. Metabolite values differ, as do reaction free energies. In this case, the maximum entropy solution obeying the constraints of Eqs (11) and (12) is the most probable solution and has stationary action.

Since the solution to Eq (10) has the same solution when the steady state extent $\xi_{j}^{ss}$ of each reaction *j* is used instead of the steady-state rate ${\dot{\xi }}_{j}^{ss}$,

$${\left[\begin{array}{l} {\left[K_{1}Q_{1}^{-1}{\left(e^{\beta \mu_{B}}\right)}^{\sum_{i}\gamma_{i,1}}\right]}^{\xi_{1}^{ss}}\\ \vdots \\ {\left[K_{Z}Q_{Z}^{-1}{\left(e^{\beta \mu_{B}}\right)}^{\sum_{i}\gamma_{i,Z}}\right]}^{\xi_{Z}^{ss}} \end{array}\right]}^{T}\cdot 1={\left[\begin{array}{l} e^{\xi_{1}^{ss}\lambda }\\ \vdots \\ e^{\xi_{Z}^{ss}\lambda } \end{array}\right]}^{T}\cdot 1,$$ (13)

this equation can be used to find the maximum entropy solution, as well. The latter can also be expressed by substituting each element for its reciprocal, which reverses the direction of each reaction such that,

$${\left[\begin{array}{cc} {\left[K_{1}Q_{1}^{-1}{\left(e^{\beta \mu_{B}}\right)}^{\sum_{i}\gamma_{i,1}}\right]}^{-\xi_{1}^{ss}}\\ \vdots & \\ {\left[K_{Z}Q_{Z}^{-1}{\left(e^{\beta \mu_{B}}\right)}^{\sum_{i}\gamma_{i,Z}}\right]}^{-\xi_{Z}^{ss}} \end{array}\right]}^{T}\cdot 1={\left[\begin{array}{l} e^{-\xi_{1}^{ss}\lambda }\\ \vdots \\ e^{-\xi_{Z}^{ss}\lambda } \end{array}\right]}^{T}\cdot 1,$$ (14)

Moreover, the difference Eqs (13)–(14) is also a maximum entropy solution. The difference vector is such that each element *j* is the difference in the thermodynamic odds of the forward and reverse reactions [25],

$$\text{Pr}(J+\xi_{+j}|J)-\text{Pr}(J+\xi_{-j}|J)={\left[K_{j}Q_{j}^{-1}{\left(e^{\beta \mu_{B}}\right)}^{\sum_{i}\gamma_{i,j}}\right]}^{\xi_{j}^{ss}}\;\;\;\;\;\;\;\;\;\;\;\;\;\;\;\;\;-{\left[K_{j}Q_{j}^{-1}{\left(e^{\beta \mu_{B}}\right)}^{\sum_{i}\gamma_{i,j}}\right]}^{-\xi_{j}^{ss}}$$ (15)

$\equiv \delta \text{Pr}(\xi_{j})$

In the limit that $\xi_{+j}$ and $\xi_{-j}$ are infinitesimal,

$$\lim_{\xi_{+j},\xi_{-j}\to 0}\left(\text{Pr}(J+\xi_{+j}|J)-\text{Pr}(J+\xi_{-j}|J)\right)=\frac{d\text{Pr(\textbackslash{}J})}{\}}d\xi ,$$ (16)

which is the time-independent change in probability taking into account both forward and reverse reactions. The chemical master equation (Eq 8) is then solved using this formulation to obtain the maximum entropy solution. The steady-state rates ${\dot{\xi }}^{ss}=[{\dot{\xi }}_{1}^{ss},...,{\dot{\xi }}_{Z}^{ss}{]}^{T}$ are any steady state rates that are solutions to the null space of the stoichiometric matrix **S**. For convenience, ${\dot{\xi }}^{ss}$ is scaled such that the uptake rate for the carbon sources, malate, and acetate have values of 1000 molecules sec^−1^. The formulation of Eq (15) is convenient as it also allows for prediction of the regulation of enzymatic reactions, as described next.

### Control and optimization

Without regulation of enzyme activity, enzyme-catalyzed reactions will result in metabolite levels at concentrations so high that the cytosol becomes glassy and diffusion drops significantly [39–42]. Control of metabolite concentrations is implemented using activity coefficients for the reactions that reflect the activity of the enzyme catalyst. The activity coefficient for any reaction *j* ranges from $\alpha_{j}=0$, where the enzyme has no activity, to $\alpha_{j}=1$, where the enzyme is fully active. For reactions that do not add or remove additional particles such that $\sum_{i}\gamma_{i,j}=0$, the activity coefficient exerts control over a reaction by scaling the reaction odds,

$$\delta \text{Pr}(\xi_{j},\alpha_{j})=\alpha_{j}(K_{j}Q_{j}^{-1}-K_{-j}Q_{-j}^{-1}).$$ (17)

While controlling metabolite concentrations may be a primary role of metabolic regulation [39,40], natural selection also requires that organisms grow fast and efficiently - using the available energy from the environment to ensure survival and compete with others. Often, this means down-regulating reactions that do not significantly increase the fitness or ability of the organism to replicate. We use a method referred to as pathway-controlled optimization (PCO) to obtain this biological goal. Next, we will briefly describe the intent of the method. For full details and examples, see [27].

Let ℐ be the set of all metabolites and let ${ℐ}_{f}$ be the set of metabolites *i* to be held at fixed concentrations ${\hat{n}}_{i}$ as boundary conditions for the system. Let ${ℐ}_{v}=ℐ\;⧵\;{ℐ}_{f}$ be the set of variable metabolites. Furthermore, let the set of all *Z* reactions be 𝒥 and let the subset of reactions corresponding to the production of biomass be 𝒢⊂𝒥, such as the reactions producing DNA, RNA, proteins, and fatty acids. We desire a steady state that maximizes biomass production, given the boundary conditions, such that the metabolites that vary cannot exceed concentrations that would cause the cytosol to become glassy. Formally, we solve the optimization problem,

$$\max\sum_{j\in 𝒢}\delta \text{Pr}(\xi_{j},\alpha_{j})$$ (18a)

subject to:

$$\frac{dn_{i}}{dt}\;=\;0\;\;\forall \;i\in {ℐ}_{v}\;,$$ (18b)

$$0\leq n_{i}\leq n_{\max}\;\;\forall \;i\in {ℐ}_{v}\;,$$ (18c)

$$n_{i}\;=\;{\bar{n}}_{i}\;\;\forall \;i\in {ℐ}_{f}\;,$$ (18d)

$$0\leq \alpha_{j}\leq 1\;\;\forall \;j\in 𝒥\;.$$ (18e)

The objective (18a) seeks to maximize the entropy through the growth reactions 𝒢 while the constraint (18b) ensures that the steady state condition is satisfied and constraint (18c) ensures that the metabolites stay within their physiological or experimentally measured values. The objective seeks a maximal entropy solution given the constraints rather than a maximum entropy solution that could be obtained without the constraints. The boundary condition for the ordinary differential equations consists of fixed boundary species such as ATP, NAD(P)H, NAD(P)+, and other cofactors. The boundary conditions are enforced by constraint (18d). Likewise, the activity coefficients are constrained to values [0,1] by constraint (18e).

The formulation of the problem is simple to express but difficult to solve. Due to the activity coefficients, the steady state constraints (18b) are nonlinear and non-convex, presenting significant challenges to optimization. Values for the reaction odds, activity coefficients, and metabolite concentrations can also vary over many orders of magnitude, which introduces additional difficulty in employing numerical methods to compute solutions. We reformulate the optimization problem to be more computationally tractable and solve the problem with an interior point solver [43] and advanced linear algebra library [44], as described in [27]. Open source code in the form of a Jupyter Notebook in Python is freely available, as described in S1 Text.

As the system approaches the optimum, the optimization surface can become flat, and the optimization can sometimes have difficulties converging. Control can specifically be an issue as some reactions in the system approach equilibrium [45] while other reactions are far from equilibrium. In these cases, some reaction free energies and their respective odds difference both approach 0.0, making control of the reactions, and hence convergence, effectively noisy. Convergence can always be obtained by adjusting either the hyperparameter controlling lower bounds on metabolite log concentrations, VarM_lbound in the source code, or the hyperparameter *Mb*, which scales the reaction free energies, as these hyperparameters manipulate the optimization surface. However, to be able to compare steady-state solutions across different redox conditions, we always used VarM_lbound=−300 and *Mb* = 1000, and in cases in which the optimizations did not converge, we adjusted the redox condition minimally by increasing or decreasing the ratio of NADP+/NADPH to be slightly above or below the corresponding target level for ${\text{KQ}}^{-1}$ (shown in the top row of Table 4). Specifically, see the top of the rightmost column in Table 4, in which case the target for the reaction odds $KQ^{-1}=10^{-3}$ but instead of using the corresponding value for the NADP+/NADPH ratio of $7.38\mathrm{\backslash time}10^{-4}$, a value of $7.57\times 10^{-4}$ was used, which corresponds to an actual $KQ^{-1}=0.975\times 10^{-3}.$ The optimizations were carried out on an Apple MacBook Pro with an Intel i9 core and 64 GB of memory.

**Table 4:** The top two rows show the correspondence between thermodynamic odds of oxidation of substrate (H_2_, Eq (24) and the ratio of concentrations of NADP+ to NADPH. The second row shows the actual ratio of concentrations used. These values are held fixed in each simulation. The lower rows show the observed ratio of various redox pairs during each simulation.

	Oxidative		Reductive
${\text{KQ}}^{-1}$	10^10^	10^9^	10^8^	10^7^	10^6^	10^5^	10^4^	10^3^
NADP+/NADPH	7383.5	738.35	73.8	7.38	0.738	7.38 $\times 10^{-2}$	7.38 $\times 10^{-3}$	7.57 $\times 10^{-4}$
Malate Growth:								
NAD+/NADH	1.44e+05	1.60e+05	3.97e+04	1.70e+05	1.60e+04	5.23e+03	2.97e+03	6.16e+02
Q/QH_2_	3.88e-10	6.50e-11	1.10e-11	4.78e-12	8.25e-13	1.44e-13	2.19e-14	3.91e-15
Fe_*ox*_/Fe_*red*_	2.70e+06	1.06e+06	4.57e+05	1.51e+05	9.55e+04	4.05e+04	1.60e+04	7.07e+03
Trdx_*ox*_/Trdx_*red*_	3.60e+02	4.78e+01	6.27e+00	8.05e-01	1.31e-01	2.14e-02	3.26e-03	5.74e-04
cyt-C_*ox*_/cyt-C_*red*_	1.71e+01	7.01	2.89e+00	1.90e+00	7.90e-01	3.30e-01	1.29e-01	5.44e-02
FAD/FADH2	5.08e+00	5.54e-01	1.92e-01	5.55e-03	3.39e-04	2.07e-05	1.02e-06	2.01e-08
Acetate Growth:								
NAD+/NADH	2.82e+05	8.32e+04	6.90e+04	8.83e+03	6.74e+03	5.20e+03	2.70e+03	2.29e+03
Q/QH_2_	2.06e-12	2.87e-10	4.31e-03	5.17e-11	1.23e-15	3.39e-14	8.30e-15	2.15e-15
Fe_*ox*_/Fe_*red*_	5.03e+06	1.76e+06	4.92e+05	1.96e+05	6.73e+04	2.98e+04	1.26e+04	5.08e+03
Trdx_*ox*_/Trdx_*red*_	5.56e+02	7.34e+01	5.80e+00	1.04e+00	1.24e-01	1.50e-02	1.92e-03	2.53e-04
cyt-C_*ox*_/cyt-C_*red*_	1.25e+00	1.47e+01	5.71e+04	6.25e+00	3.05e-02	1.60e-01	7.92e-02	4.03e-02
FAD/FADH2	9.54e+02	1.11e+04	1.99e+02	2.49e+03	3.90e+03	6.23e+03	1.56e+04	2.40e+04

Finally, a few important caveats to the PCO method should be noted. While the method makes physical intuitive sense and reproduces known regulatory phenomena for those cases in which direct experimental data is available [27], the general lack of direct experimental assays of regulation prevents more extensive testing and analysis of the method. Furthermore, the PCO method cannot distinguish between reduced activity due to allosteric interactions, post-translational modifications of enzymes, or changes in enzyme expression. While the PCO method used the maximum entropy solution as input here, the method itself does not depend on using the maximum entropy solution. Also, the method uses observed or estimated metabolite concentrations as the allowed upper limit for predicting concentrations. Biological species will depend on metabolite solubilities and solvent viscosities instead of specific concentrations for learning regulation.

### Metabolic model

The model consists of 252 reactions and 253 metabolites. Reaction pathways include those important for *R. rubrum* photoheterotrophic growth, including all of the reactions of central metabolism and the ethylmalonyl-CoA pathway for ethanol/acetate assimilation. Secondary metabolism included reactions for amino acid and nucleic acid synthesis, folate metabolism, S-adenosyl methionine metabolism, protein synthesis, DNA synthesis, RNA synthesis, and degradation. Protein synthesis, RNA synthesis, and DNA synthesis were modeled assuming that each macromolecular species (protein, RNA, and DNA) consisted of equal fractions of the constituent monomers, amino acids, ribonucleic acids, and deoxyribonucleic acids, respectively. The growth reaction for biomass included protein synthesis, DNA, RNA, and fatty acid synthesis according to the relative abundances measured experimentally under fumarate growth conditions [19], as described below. The metabolic model was developed and curated using Pathway Tools [46] and stored as a BioCyc pathway genome database [47]. The model is available in JSON format and executable in a python Jupyter notebook as described in S1 Text.

Each reaction was mass-balanced. Consequently, only reactions that could be implemented using specific molecular species, and not compounds or classes of chemicals, were used in the model. This allowed for mass action kinetics, as described above, to be used. Of the total metabolites, 228 of the metabolites are free variables, and 25 metabolites are fixed as boundary conditions. The rank of the stoichiometric matrix is 236. The set of fixed metabolites (boundary conditions) and their concentrations are listed in Table A in S1 Text. The boundary metabolites were either environmental nutrients or the metabolic model did not include de novo synthetic pathways for these compounds. Allowing these metabolites to be variable would cause the stoichiometric matrix to become singular. In addition, the standard free energy of reaction for the ATP synthesis reaction was adjusted to account for a 10-fold driving force due to the proton gradient across the cytoplasmic membrane.

Each reaction is modeled using mass action kinetics, as described above. Equilibrium constants for the reactions were determined using the eQuilibrator API software, version 0.4.0 [48], except where noted in S1 Text. A pH of 7.0 and ionic strength of 0.15 M were used throughout. The reference free energies of reaction account for the multiple charge states of ions present at the specified pH [49,50].

The steady-state solutions were constrained to replicate the experimentally observed ratios of DNA:RNA:protein during growth on malate obtained by McCulley *et al*. of 1.0: 2.9: 44.1 [19], which agree with those measured in this work for malate (Table 5). For lipid composition, we assumed that fatty acid content could be used as a proxy for lipid content. The fatty acid mass percentage of a *R. rubrum* cell is 15%. We further assumed that palmitate could be used to estimate fatty acid content. Palmitate has a molecular mass of 256.42 Da. Accordingly, the total macromolecule mass estimate relative to DNA is DNA + RNA+ Protein + FA mass = 1.0 + 2.9 + 44.1 + x such that the lipid mass percentage x was estimated from 0.15 = x / (1.0 + 2.9 + 44.1 + x), yielding x = 8.47%.

**Table 5:** (Left) Experimentally measured elemental composition of cell biomass and estimated oxidation state of carbon from the elemental formula. (Right) Experimentally measured mass ratios of macromolecules relative to DNA. Elemental analysis sources: ^*a*^ elemental composition of *R. palustris* biomass when grown on malate from Carlozzi and Sacchi [67]; ^*b*^ average elemental composition of *Rhodobacter sphaeroides* from Waligórska *et al*. [68], ^*c*^ average elemental composition of *Rhodospirillum rubrum* growth on acetate from Favier-Teodorescu, *et al*. [51]; ^*d*^ measurements for *R. rubrum* in this study.

	Measured	Carbon	Measured Macromolecular Ratios ^*d*^					
Substrate	Biomass	Ox. State	DNA	RNA	Protein	Lipid	PHB	Chromatophores
Malate	C_4_H_7.20_O_1.52_N_0.72_^*a*^	-0.50	1.00	2.96	39.98	9.40	0.07	0.42
	C_4_H_7.56_O_2.04_N_0.56_^*b*^	-0.45						
Acetate	C_4_H_6.94_O_1.57_N_0.63_^*c*^	-0.48	1.00	2.51	35.25	30.13	0.73	0.41
EtOH/CO_2_	C_4_H_7.03_O_2.03_N_0.61_^*d*^	-0.29	1.00	2.98	36.59	18.38	4.20	0.37
Butyrate/CO_2_	C_4_H_7.12_O_2.05_N_0.65_^*d*^	-0.27	1.00	2.44	31.32	9.06	9.66	0.42

The steady-state solutions were constrained by incorporating these amounts of the respective macromolecules into biomass in the form of a cell monomer, using the following pseudo-chemical reaction equation,

1.0DNA+2.9RNA+44.1protein+8.5lipid⇌1.0cell monomer. (19)

While the ratio of DNA:RNA:protein:lipid will differ experimentally as a function of the nutrient, using a fixed value calibrated to malate growth allows for the comparison of the energetics and dynamics of growth for the different nutrients for the same growth process. Subsequently, for comparison of modeling to experimental results, the macromolecule content and elemental composition of *R. rubrum* growing on various substrates were measured as detailed in the experimental methods section.

*Predicted Biomass elemental composition.* The predicted elemental composition of the modeled biomass formation is then found by the following procedure. The overall chemical equation for the cell is determined from the rate of production and consumption of the fixed (boundary) metabolites, normalized to a rate of malate or acetate consumption of 1000 molecules sec^−1^. However, if a normalized rate is less than 1/100th of the rate of malate, then it is not included in the overall chemical reactions. Using the overall reaction from a simulation as an example, the chemical equation is,


$$2.2\text{THF}+1.0\;5,10\text{-MTHF}+0.06\;5\text{-MTHF}+0.08\;{\text{SO}}_{\text{4}}+1.85\;\text{ABP }+18.5P_{i}$$

$$+10\;C_{4}H_{6}O_{5}+5.32\;{\text{NH}}_{3}+0.7\;\text{NAD}+\;+30.7\;\text{NADPH}+0.1\;{\text{CO}}_{2}$$

$$\to 3.3\;\text{N10-fTHF}+4.06\;{\text{P}}_{i,2}+\text{biomass}+31.4\;\text{NADP}+$$

First, the internal metabolites except phosphate are dropped such that the overall equation is,

$$0.7\;\text{NAD}+\;+19.2{\text{P}}_{i}+10\;{\text{C}}_{4}{\text{H}}_{6}{\text{O}}_{5}+5.32\;{\text{NH}}_{3}+30.7\;\text{NADPH}+0.1\;{\text{CO}}_{2}\;\to \text{biomass}+31.4\;\text{NADP}+.$$ (20)

Next, Eq (20) is simplified by removing the redundancy of having both NAD+ and NADP+ redox components by adding to the chemical equation,

$$0.7\;\text{NADP}+\to 0.7\;\text{NAD}+\;+0.7\;{\text{P}}_{i},$$ (21)

and canceling like terms. The overall chemical equation becomes,

$$18.5{\text{P}}_{i}+10\;{\text{C}}_{4}{\text{H}}_{6}{\text{O}}_{5}+5.32\;{\text{NH}}_{3}+30.7\;\text{NADPH}+0.1\;{\text{CO}}_{2}\;\to \text{biomass}+30.7\;\text{NADPH}+.$$ (22)

To calculate the CHON biomass elemental composition, phosphate is dropped, NADPH is replaced by H_2_, and NADP+ is replaced by a variable amount of H_2_O. Next, ‘biomass’ is substituted by the variable composition formula C_4_H_*u*_O_2.04_N_*z*_, where the value of 2.04 for oxygen was determined from experimental elemental analysis of biomass (for biomass grown on acetate, a value of 1.57 was used [51]) (Table 6). The resulting chemical equation,

**Table 6:** Predicted elemental biomass composition (bold) estimated from overall chemical reactions for growth on malate. The color of blue or green for the overall cellular reaction indicates that the cell operates in a reductive or oxidative cycle, respectively, as shown in Fig 1. Estimates were obtained from simulations under oxidizing, neutral and reducing redox conditions, according to the concentrations of NADP+:NADPH being held at the thermodynamic odds (Eq 24) of 10^10^, $9.9\;\times \;10^{6}$ and 10^3^, respectively. The rows labeled *Simulation* are those from the optimization for growth of the metabolic model while those labeled *Estimate* are estimates of the biomass elemental composition using the stoichiometry found in the model. The grey highlighted rows are for those compounds in the overall chemical equation for the metabolic model that are not used in estimating the elemental composition of the biomass. Abbreviations: ABP: adenine-3,5-bisphosphate; THF: tetrahydrofolate; 5,10-MTHF: 5, 10 methylenetetrahydrofolate; N10-fTHF: N10-formyltetrahydrofolate; P_*i*,2_: diphosphate.

State (Eq 24)	Overall Reaction			Biomass C State
**oxidized**	2.52 THF + 1.49 5,10-MTHF + 0.05 5-MTHF +			
$\left(10^{10}\right)$	0.07 SO_4_ + 2.18 ABP + 21.37 P_*i*_ +	→	4.06 N10-fTHF + 3.08 P_*i*,2_ +	
*Simulation*:	10 C_4_H_6_O_5_ + 3.76 NH_3_ + 19.46 NADPH		biomass + 19.46 NADP+ + 4.52 CO_2_	
*Estimate*:	10 C_4_H_6_O_5_ + 3.76 NH_3_ + 19.46 H_2_ &	→	8.9 **C**_4_**H**_7.27_**O**_2.04_**N**_0.42_ + 22.9 H_2_O + 4.5 CO_2_	-0.48	
**neutral** $\left(10^{6.99}\right)$	2.2 THF + 1.0 5,10-MTHF + 0.06 5-MTHF + 0.08 SO_4_ + 1.85 ABP + 18.5 P_*i*_ +	→	3.3 N10-fTHF + 4.06 P_*i*,2_ +	
*Simulation*:	10 C_4_H_6_O_5_ + 5.32 NH_3_ + 30.7 NADPH + 0.1 CO_2_		biomass + 30.7 NADP+	
*Estimate*:	10 C_4_H_6_O_5_ + 5.32 NH_3_ + 30.7 H_2_ + 0.1 CO_2_	→	10 **C**_4_**H**_7.7_**O**_2.04_**N**_0.53_ + 29.6 H_2_O	-0.50	
**reduced** $\left(10^{3}\right)$	1.43 THF + 0.79 5,10-MTHF + 0.18 5-MTHF + 0.23 SO_4_ + 1.71 ABP + 9.57 P_*i*_ +	→	2.4 N10-fTHF + 3.83 P_*i*,2_ +	
*Simulation*:	10 C_4_H_6_O_5_ + 20.50 NH_3_ + 187.2 NADPH + 67.61 CO_2_		biomass + 187.2 NADP+	
*Estimate*:	10 C_4_H_6_O_5_ + 20.50 NH_3_ + 187.2 H_2_ + 67.61 CO_2_	→	26.9 **C**_4_**H**_8.74_**O**_2.04_**N**_0.76_ + 130.34 H_2_O	-0.60


$$10\;{\text{C}}_{4}{\text{H}}_{6}{\text{O}}_{5}+5.32\;{\text{NH}}_{3}+30.7\;{\text{H}}_{2}+0.1\;{\text{CO}}_{2}\;\to x\;C_{4}H_{u}O_{2.04}N_{z}+y\;H_{2}O,$$

can be solved for the values of x, y, z, and u by balancing carbon, oxygen, nitrogen, and hydrogen, respectively [18].

### Experimental quantification of macromolecules and element ratios

*R. rubrum* ATCC 11170 was grown as previously reported in 500 ml of Ormerod’s minimal media in sealed Roux bottles at 30 degrees Celcius under 2000 lux incandescent illumination in biological triplicate. Cultures were supplemented with 85 mM ethanol and 0.1% sodium bicarbonate, 10 mM sodium butyrate with 0.1% sodium bicarbonate, 20 mM DL-malic acid, or 20 mM acetic acid and flushed with nitrogen to establish anaerobic conditions [23]. Cells were collected by centrifugation during exponential growth at an Optical Density of 660 nm of ~0.80.

*Biomass elemental analysis.* Pellets from 200 ml culture were washed three times with ultrapure water to remove compounds present in the residual media and lyophilized to dryness. Dry cell mass (50 mg) was analyzed on a VarioEL Cube elemental CHNS/O analyzer (Elementar, New York) following the manufacturer’s protocols at the DOE EMSL laboratory.

*Dry cell weight.* Pellets from 50 ml culture were lyophilized to dryness in pre-weighed test tubes and measured gravimetrically to determine the dry cell weight per volume and density of culture at time of cell collection for calculating macromolecules by mass ratio.

*Lipid analysis.* Total extractable hydrophobic compounds (lipids, photosynthetic pigments, poly-hydroxy butyrate (PHB)) were measured by organic extraction following a modified version of the Folch method [52,53]. 50 ml cell culture was collected by centrifugation in chloroform-treated plastic tubes. Pellets were lyophilized to dryness and extracted using 2 mL chloroform:methanol (2:1 [v/v]). After centrifugation at 4000 RCF for 5 minutes, the supernatant was transferred to a new glass tube, and the remaining pellet was re-extracted twice more with 2 mL chloroform:methanol as before. Extracts were pooled and washed with 0.9% KCl at a ratio of chloroform:methanol:0.9% KCl of 8:4:3 [v/v/v]. After the addition of KCl, samples were vortexed, centrifuged, and the upper aqueous phase aspirated off. This was performed three times, and afterward, the lower organic phase was collected and transferred to a pre-weighed glass lyophilization vial. Solvent was removed by evaporation under a stream of nitrogen at 50 degrees Celcius and total extractable hydrophobic compounds were measured gravimetrically. To determine total lipids, the total measured PHB and photosynthetic pigment (chromatophores) were subtracted out as detailed below.

The total PHB present in the cell pellet from 20 ml of cell culture was quantified using the method of Slepecky and Law [54]. Similarly, total photosynthetic pigment (chromatophores) was quantified using the method of van der Rest and Gingras by UV-Vis quantitation of extracted chromatophores in acetone [55].

*Protein quantification.* Cells from 5 ml culture were resuspended in 700 μl EDTA-free buffer (0.1 M Tris-HCl, 2% SDS, pH 8.0), then sonicated for 3 minutes with 1 second on and 5 second off pulses. Samples were centrifuged for 3 minutes at 5000 RCF and the supernatant was used in BCA assays (Pierce, Thermo Scientific) following manufacturer’s instructions. All BCA assays were performed in technical duplicate on the biological replicates.

*DNA/RNA quantification.* Cells from 5 ml culture were immediately treated with Qiagen RNAprotect upon cell collection by centrifugation and stored at 4 degrees Celcius until analyzed. RNAprotect was removed by centrifugation, and cells were resuspended in 700 μl of 0.1 M Tris-HCl, 2% SDS, 0.1 M EDTA, pH 8.0. Cells were sonicated as above and centrifuged for 3 minutes at 5000 RCF. HPLC analysis was performed by the method of Dell’anno *et al*. [56] with the following modifications. Supernatants were applied to an anion exchange column (TSKgel DEAE-5PW, TOSOH Bioscience) connected to a Shimadzu Prominence HPLC with UV detection at 260 nm. DNA and RNA were eluted from the column on a 0.1–1 M KCl gradient in 20 mM K-phosphate buffer with 5 M urea, pH 6.8, over 24 minutes. DNA and RNA concentrations were calculated from peak areas compared to standard calibration curves generated using DNA from salmon testes (Sigma-Aldrich) and RNA from baker’s yeast (Sigma-Aldrich).

## Results

**Overview.** As discussed in the Introduction, we are interested in the redox conditions governing the assimilation of malate and acetate relative to CO_2_ production or consumption and how the metabolism of *R. rubrum* adjusts for differing internal redox conditions by dissipating reductant, if necessary. To do so, we constructed a mass action thermodynamic and kinetic model of *R. rubrum’s* metabolism that included 253 metabolites and 252 reactions, as summarized in Fig 2, including pathways for amino acid, nucleotide, RNA, DNA, and lipid synthesis, photosynthesis, and the electron transport chain. The model of photosynthesis and the electron transport chain follows that outlined by Klamt, *et al*. [9] for purple nonsulfur bacteria based on experimental studies [10].

Malate and acetate were used as organic carbon sources. The chemical equation for each of the main reactions for CO_2_ assimilation are shown in Table 3 and Fig 2 (orange boxes). The routes of CO_2_ assimilation included the CBB cycle, the ethylmalonyl-CoA pathway, the ferredoxin-dependent 2-oxoglutarate synthase reaction, the isocitrate dehydrogenase reaction, the ferredoxin-dependent pyruvate synthase reaction, and the phosphoenolpyruvate carboxykinase reaction. The malic enzyme reaction (NAD+-Malic Enzyme, non-oxaloacetate decarboxylating, E.C.1.1.1.37), PEP carboxytransphosphorylase (E.C.4.1.1.38), and PEP carboxylase (E.C.4.1.1.32) were not included in the model because several studies have shown them to be negligibly active under the study conditions [21,57,58].

**Fig 3:**
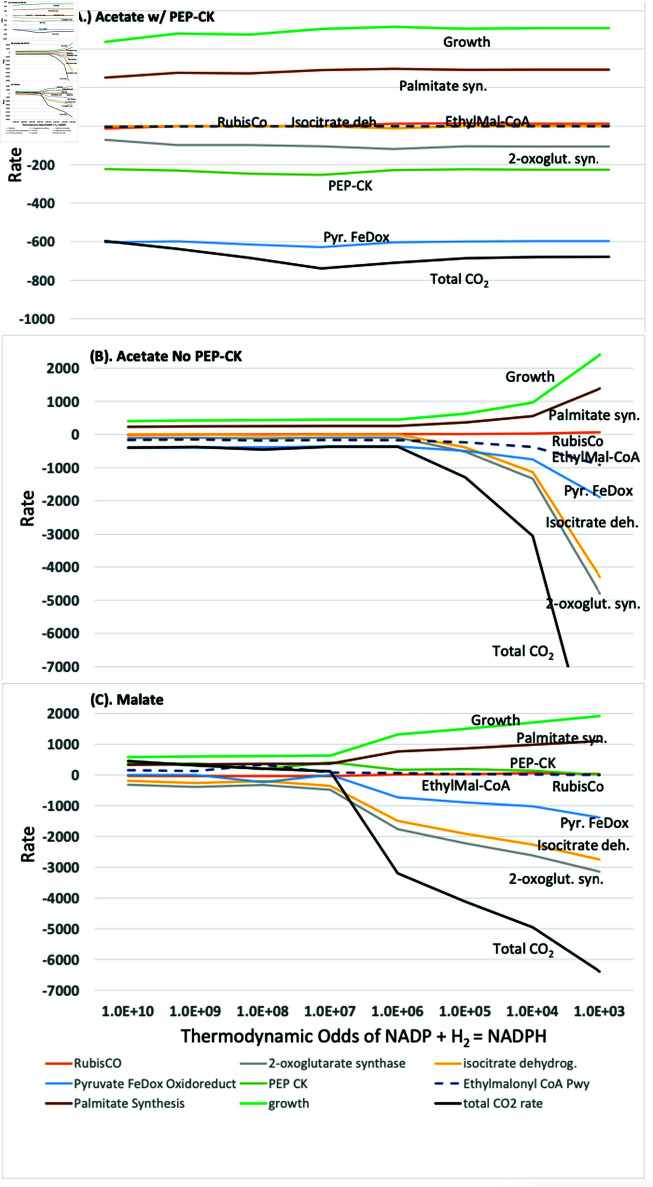
Plots of growth rate, palmitate (fatty acid synthesis) rate, and rates of CO_2_ flux at the Ethylmalonyl-CoA pathway, RubisCO, 2-oxoglutarate synthase, isocitrate dehydrogenase, pyruvate ferredoxin oxidoreductase, phosphoenolpyruvate carboxykinase (PEPCK) as a function of the thermodynamic odds (${\text{KQ}}^{-1}$) of NADP+:NADPH. A thermodynamic odds (${\text{KQ}}^{-1}$, Eq (24) of 10^−3^ indicates that ratio of products to reactants (Q) in the chemical reaction NADPH ⇌ NADP+ + H_2_ is 1000-fold higher compared to the equilibrium ratio (K), assuming that the hydrogen concentration is at the reference value of 1 M in Eq (24).

**Internal Redox State and CO**_**2**_ **production rates.** The fluxes through these reactions were analyzed across seven orders of magnitude of the thermodynamic odds for NADP/NADPH, which represents the odds ratio of redox carrier pair concentrations. The fluxes as a function of the odds are shown in Fig 3. The values on the horizontal axis (x-axis) represent the thermodynamic odds for the substrate oxidation reaction,

$${\text{NADP+ + H}}_{2}⇌\text{NADPH}\;\;\;\;\;\;\Delta {\text{G}}^{\circ }=-35.0\text{kJ/mol},$$ (23)

such that the thermodynamic odds is given by,

$$KQ^{-1}=e^{-\Delta G^{\circ }/RT}\cdot \frac{\left[NADP+][H_{2}\right]}{\left[NADPH\right]},$$ (24)

which in this case the substrate is molecular hydrogen and [H_2_] is 1 M. A ${\text{KQ}}^{-1}$ value of 1.0 indicates the reaction is at equilibrium, whereas at a thermodynamic odds of 10^4^, the oxidation of the substrate H_2_ is favored by 10000-fold. The value of 1 M for [H_2_] is used as a reference value since it allows comparison to the standard hydrogen electrode, a reference for half-cell potential reactions, and the basis for the standard scale of oxidation-reduction potentials.

The correspondence between the thermodynamic odds for oxidation (Eq 24) and ratios of NADP+ and NADPH concentrations are shown in Table 4. Note that at a thermodynamic odds of 10^4^, the NADP+/NADPH concentration ratio is approximately 10^−2^, which is the generally accepted value for the average of the concentration ratio and thus redox poise of the cell. However, the concentration ratios shown in Table 4 cannot be directly compared to experimentally measured values because the concentrations used in the simulation model are those of the unbound metabolic species since it is the unbound concentrations that determine the overall thermodynamics of the reaction. In contrast, experimental assays typically measure whole-cell populations, including both enzyme-bound and unbound species. As the thermodynamic odds for oxidation of substrate increase from 10^4^ toward 10^7^ the NADP+/NADPH ratio approaches unity indicating that the cell redox poise is relatively more oxidizing than typically accepted values. As thermodynamic odds for oxidation decrease from 10^4^ toward 1.0 the NADP+/NADPH ratio becomes increasingly smaller indicating that the cell redox poise is relatively more reducing than typically expected values.

For both growth on malate and acetate, several similar trends stand out in Fig 3 regarding growth and CO_2_ production rates. First, as the redox level moves away from typical values (thermodynamic odds of oxidation of 10^4^) towards more oxidative conditions, the overall level of CO_2_ assimilation decreases, as expected. For malate, the trend is significant in that the CO_2_ assimilation rate (per 1000 units of malate) changes from a net uptake rate of 6394 units to a net production rate of 451 units as the NADP+/NADPH ratio (oxidative potential) increases from $7.57\;\times \;10^{-4}$ to $7.38\;\times \;10^{3}$. The trend is similar for acetate, with an exception noted below due to the activity of phosphoenolpyruvate carboxykinase (PEP-CK).

Second, CO_2_ assimilation through the CBB cycle was close to zero under any NADP+/NADPH ratio. This is consistent with experimental observations using ${\text{C}}^{13}$ metabolic flux analysis by McCulley, *et al*. [19] on *R. rubrum* and McKinlay, *et al*. on *Rhodopseudomonas palustris* [12]. Growth increased from near 500 units to approximately 2000 units on both malate and acetate as thermodynamic odds moved from oxidative (>10^4^) toward reductive conditions (<10^4^), as did fatty acid synthesis. This is expected because as the thermodynamic odds move toward 1.0, the available NADPH relative to NADP+ increases (Table 4), and the free energy barrier for the reduction of substrate to biomass decreases.

Finally, under relatively reductive conditions, the primary routes for CO_2_ assimilation were predicted by the model to be the reactions of the reductive TCA cycle and pyruvate metabolism. Under reductive conditions ($\text{odds}=10^{3}$, NADP+/NADPH $=7.57\times 10^{-4}$), the flux reached 3150 units (malate) and 4803 units (acetate), as observed at the 2-oxoglutarate synthase reaction. In contrast, the reductive TCA cycle had a flux near zero under highly oxidative conditions (odds of oxidation 10^10^, NADP+/NADPH  = 7383.5) in all cases. Even under these highly oxidative conditions, the TCA cycle did not operate in the fully oxidative direction.

Moreover, under all conditions examined, net NADPH consumption, rather than production, was the rule (*vide infra*, Tables 6 and 7). While the forward reaction in Eq (23) is favored, the coupling of redox and anabolic reactions to ATP hydrolysis and other processes makes NADPH consumption more favorable.

**Table 7:** Predicted elemental biomass composition (bold) estimated from overall chemical reactions for growth on acetate. The color of blue for the overall cellular reaction indicates that the cell operates in a reductive cycle, as shown in Fig 1. Estimates were obtained from simulations under oxidizing, neutral and reducing redox conditions, according to the concentrations of NADP+:NADPH being held at the thermodynamic odds (Eq 24) of 10^10^, 10^7^ and 10^3^, respectively. The rows labeled *Simulation* are those from the optimization for growth of the metabolic model while those labeled *Estimate* are estimates of the biomass elemental composition using the stoichiometry found in the model. The grey highlighted rows are for those compounds in the overall chemical equation for the metabolic model that are not used in estimating the elemental composition of the biomass. Abbreviations: ABP: adenine-3,5-bisphosphate; THF: tetrahydrofolate; 5,10-MTHF: 5, 10 methylenetetrahydrofolate; N10-fTHF: N10-formyltetrahydrofolate; P_*i*,2_: diphosphate.

State (Eq 24)	Overall Reaction			Biomass C State
**oxidized** (10^10^)	2.5 THF + 1.5 5,10-MTHF + 0.05 5-MTHF + 0.07 SO_4_ + 2.2 ABP + 20.6 P_*i*_ +	→	4.0 N10-fTHF + 3.1 P_*i*,2_ +	
*Simulation*:	10 C_2_H_4_O_2_ + 4.23 NH_3_ + 14.20 NADPH + 3.95 CO_2_		biomass + 14.20 NADP+	
*Estimate*:	10 C_2_H_4_O_2_ + 4.23 NH_3_ + 14.20 H_2_ + 3.95 CO_2_	→	5.99 **C**_4_**H**_7.37_**O**_1.57_**N**_0.71_ + 18.48 H_2_O	-0.52
**neutral** (10^7^)	0.85 THF + 0.05 5,10-MTHF + 0.04 5-MTHF + 0.05 SO_4_ + 0.59 ABP + 21.06 P_*i*_ +	→	0.95 N10-fTHF + 10.37 P_*i*,2_ +	
*Simulation*	10 C_2_H_4_O_2_ + 3.81 NH_3_ + 15.26 NADPH + 3.65 CO_2_		biomass + 15.26 NADP+	
*Estimate*	10 C_2_H_4_O_2_ + 3.81 NH_3_ + 15.26 H_2_ + 3.65 CO_2_	→	5.91 **C**_**4**_**H**_**7.77**_**O**_**1.57**_**N**_**0.64**_ + 18.00 H_2_O	-0.68
**reduced** (10^3^)	4.51 THF + 0.09 5,10-MTHF + 0.22 5-MTHF + 0.29 SO_4_ + 3.05 ABP + 19.2 P_*i*_ +	→	4.83 N10-fTHF + 11.90 P_*i*,2_ +	
*Simulation*	10 C_2_H_4_O_2_ + 20.5 NH_3_ + 255.16 NADPH + 106.11 CO_2_		biomass + 255.16 NADP+	
*Estimate*	10 C_2_H_4_O_2_ + 20.5 NH_3_ + 255.16 H_2_ + 106.11 CO_2_	→	31.53 **C**_**4**_**H**_**7.82**_**O**_**1.57**_**N**_**0.65**_ + 182.66 H_2_O	-0.68

**Comparison to experiment.** Phototrophic growth under anaerobic conditions would naively be expected to result in a reductive environment inside the cell due to the initial generation of NADPH as a result of photosynthesis. In this case, conversion of NADPH to NADP+ in downstream reactions would be favored, reflective of thermodynamic odds approaching 1 on the right side of the x-axis of Fig 3. On the contrary, the simulation results agree with inferences based on experimental observations made using ${\text{C}}^{13}$ metabolic flux analysis that the internal redox environment *in vivo* is relatively oxidized. Indeed, the simulation results are consistent with the ${\text{C}}^{13}$ metabolic flux analysis data only in the relatively oxidative range of NADP+/NADPH ratios from the center to left-hand side of the x-axis in Fig 3 (thermodynamic odds of oxidation $\geq 10^{7}$).

Of course, caveats in both the model and the metabolic flux analysis need to be considered. In metabolic flux analysis, a model is used to generate flux distributions, which in turn are used to generate predicted isotope labeling patterns. The predicted isotope labeling patterns are then compared to experimentally measured isotope patterns, and the best-fitting computational isotope pattern implicates the analogous flux distribution. The inference can be very sensitive to the computational model that is used [59]. The models used in the study by McCully, *et al*. in [19] were understandably relatively small and also included the first few reactions of the oxidative pentose phosphate pathway [19], which are not likely in the species. In addition, it was assumed that flux was unidirectional from isocitrate to 2-oxoglutarate for all models. In the simulation model, isocitrate flux was in the reductive direction (2-oxoglutarate to isocitrate) in all cases. Thus, clarification of this issue is an open question.

However, a direct comparison of the 252 reaction fluxes from the simulation model and the 64 reactions of the MFA model for malate of McCulley, *et al*. is difficult because there are only 18 reactions that overlap in the two models (not counting the malate uptake reaction). This is due to the limited size of the MFA model and because many of the 64 MFA reactions are composite reactions representing complex pathways of secondary metabolism that had no direct counterpart in the detailed simulation model. Of the 18 reactions that are in both models, at a thermodynamic odds of 10^7^, 10 of these have flux in the same direction, and these 10 reactions are all in central metabolism. Of the 8 reaction fluxes that do not agree, four are in the pentose phosphate pathway/Calvin-Benson-Bassham cycle, one is the isocitrate dehydrogenase reaction, one is in secondary metabolism (serine hydroxymethyltransferase), one is the malate synthase reaction, and one is the citrate synthase transformation. These reactions for each model are shown in S1 Table.

**TCA cycle.** For the comparison to the metabolic flux analysis model, we evaluated the thermo-kinetic models containing both the oxidative and reductive versions of the tricarboxylic acid (TCA) cycle, both separately and together. Since the ATP-dependent citrate lyase, a key enzyme of the reductive TCA cycle, is missing from *R. rubrum*, this has led some to speculate that the reductive TCA cycle may not operate in *rubrum* [60]. Support for this hypothesis was the lack of detection of citrate and acetate in *in vivo* assays even though earlier physiological and enzymatic evidence suggested a functioning, albeit low-flux reductive TCA cycle in *R. rubrum* [8,61]. However, due to the transient nature of non-equilibrium concentrations, a lack of observation cannot be used to draw conclusions. *R. rubrum* does contain the ATP-independent citrate lyase and acetate CoA ligase enzymes, which together carry out the same transformation as ATP-dependent citrate lyase [62,63]. Our model of the reductive TCA cycle includes the ATP-independent citrate synthase used in the oxidative TCA cycle as well as both citrate lyase and acetate CoA ligase reactions.

Each version of the TCA cycle was complemented with the appropriate oxidative/reductive version of the pyruvate to acetyl-CoA reaction: either pyruvate synthase for the reductive TCA cycle or pyruvate dehydrogenase for the oxidative TCA cycle. The reductive and oxidative processes differ in both the reactions for converting pyruvate to acetyl-CoA and 2-oxoglutarate to succinyl CoA, in which ferredoxin (Fd) is used for the reductive processes and NAD+ for the oxidative processes. The ferredoxin redox pairs are favored under reducing conditions since their reduction potential is slightly more favorable for carrying out reductive reactions,

$$2{\text{FD}}_{red}+\text{NAD}+\;\;⇄\;2{\text{FD}}_{\alpha x}+\text{NADH}\;\Delta G=-15.5\;KJ/\text{mol}\;\text{at}\;\text{pH}7.0.\;$$ (25)

The results were not qualitatively different in that, in all three cases, the TCA cycle ran similarly for each growth condition using both malate and acetate as the primary carbon source. The only quantitative difference was that the TCA cycle carried a slightly higher net flux when the reactions of the reductive TCA cycle were used under reductive conditions instead of the reactions of the oxidative TCA cycle. This illustrates a common misconception regarding cellular thermodynamics, that many reactions are not reversible unless a specific enzyme is present. Thermodynamics determines the net directionality of a reaction - always - and the role of the catalyst is to reduce the transition state barrier. Enzymes associated with specific directions of reactions, such as NAD-dependent 2-oxoglutarate dehydrogenase and ferredoxin-dependent 2-oxoglutarate synthase, are selected by nature because they reduce thermodynamic costs for specific conditions. However, this does not mean the reaction can go only in one direction. Given enough reactant, these seemingly one-way reactions can be reversed, as has been recently observed whereby the oxidative TCA cycle runs in reverse in the appropriate conditions [64,65]. Since the TCA cycle mainly operated in the reductive direction across the range of redox conditions (Fig 3) regardless of which enzymes were used, the results below pertain to the model using the reductive TCA cycle and the pyruvate ferredoxin oxidoreductase.

**Phosphoenolpyruvate carboxykinase.** In support of the hypothesis that the internal environment is not highly reduced during growth on malate is the observation that the conditions in the model in which the flux through phosphoenolpyruvate carboxykinase (PEP-CK) best agrees with the experimental MFA data of McCulley, *et al*. is when the thermodynamic odds of oxidation in the reference reaction (Eq 24) is approximately 10^7^ or above (Fig 3). In both the experimental MFA fluxes and in the model under oxidative conditions during growth on malate, the reversible PEP-CK reaction had significant flux in the direction of conversion of oxaloacetate to phosphoenolpyruvate,


$$\text{oxaloacetate}+\text{GTP}⇄\;\text{PEP}+{\text{CO}}_{2}+\text{GDP},$$

or alternately,


$$\text{oxaloacetate}+\text{ATP}⇄\;\text{PEP}+{\text{CO}}_{2}+\text{ADP},$$

Under relatively more reductive conditions in which the odds of oxidation in the reference reaction were below 10^7^, the reaction in the model had no significant flux.

In contrast, during growth on acetate, the PEP-CK reaction had flux in the opposite direction, assimilating CO_2_ and channeling carbon from phosphoenolpyruvate into oxaloacetate and then malate. The ratio of GTP:GDP and ATP:ADP remained relatively stable throughout the redox range, allowing the PEP-CK reaction to remain favorable for CO_2_ assimilation. Consequently, CO_2_ assimilation through PEP-CK did not decrease proportionately with a decrease in reductive power. Yet, significant reducing power is required to cycle the oxaloacetate produced by PEP-CK to malate and through the reductive TCA cycle into 2-oxoglutarate and pyruvate for biosynthetic purposes. Interestingly, during growth on acetate, the ethylmalonyl-CoA anaplerotic pathway did not produce malate and succinate under the simulation conditions. The ethylmalonyl-CoA pathway is thought to be essential for acetate growth for *R. rubrum*. (Unfortunately, previous metabolic flux analysis studies for growth on acetate [12] were carried out before it was known that *R. rubrum* used the ethylmalonyl-CoA pathway and did not contain the glyoxylate bypass.) Therefore, the lack of functionality of the ethylmalonyl-CoA pathway in our simulations required further analysis.

Turning off the PEP-CK reaction alleviates the situation: CO_2_ assimilation occurs primarily through the ethylmalonyl-CoA pathway during growth on acetate. Empirically, it has been found that high ATP concentrations, which would be expected during phototrophic growth, act to unidirectionally inhibit the PEP-CK reaction in the direction of oxaloacetate formation [66]. Consequently, the PEP-CK reaction was turned off in subsequent models used for acetate growth.

**Ethylmalonyl-CoA pathway.** Using the ethylmalonyl-CoA pathway instead of the PEP-CK reaction to anapleroticaly produce malate and succinate during growth on acetate may be favorable because the ethylmalonyl-CoA pathway is more sensitive to redox conditions. The overall reaction for the ethylmalonyl-CoA pathway in *R. rubrum* is,

$$3\;\text{acetyl}-\mathrm{CoA}+\mathrm{NADH}+\mathrm{NADPH}+\mathrm{FAD}+{\mathrm{CO}}_{2}+H_{2}O\;\;⇄\;\text{malate}+\text{propanoyl}-\text{CoA}+\text{NAD}++\text{NADP}++{\text{FADH}}_{2}+2\text{CoA}\text{.}$$ (30)

Two of the final steps in the pathway convert glyoxylate, water, and acetyl-CoA to malate and CoA. This is carried out in two steps, with (S)-Malyl CoA as an intermediate. However, we observed that the (single-step) malate synthase reaction, glyoxylate + acetyl-CoA + H_2_O ⇌ malate + CoA, can be substituted for the former with identical results. We were not able to utilize both sets of reactions (one set directly making malate and the other set making malyl-CoA as an intermediate from glyoxylate) at the same time, as doing so caused the ethylmalonyl-CoA pathway to go to equilibrium, stopping acetate assimilation. The model still grew when both sets of reactions were used, but by strictly CO_2_ assimilation processes other than the ethylmalonyl-CoA pathway.

The overall reaction of the ethylmalonyl-CoA pathway is enhanced under reductive conditions (odds of oxidation < 10^4^ in Eq 24). The lone oxidative reactant FAD is more than offset by the co-reactant NADPH in that the reaction FAD + NADPH ⇌ FADH_2_ + NADP+ favors the products by approximately 23 kJ/mol at pH 7.0. The product propanoyl-CoA is then further metabolized to succinate with an overall reaction for the pathway,

$$\text{propanoyl}-\text{CoA}+\text{ATP}+{\text{H}}_{2}{\text{CO}}_{3}\;⇄\;\text{succinate}+\text{CoA}+\text{ADP}+\text{pi}\text{.}\;$$ (31)

Despite the experimentally-observed requirement for the presence of the ethylmalonyl-CoA pathway for growth on acetate, this pathway was not the major pathway of acetyl-CoA assimilation. We tested whether this pathway could become the major assimilation pathway for acetyl-CoA for both acetate and malate growth by incrementing the standard free energy change for the ATP synthase reaction by 4·RTlog50 such that ATP formation was driven by a 50-fold change in pH instead of a 10-fold change. Flux through the ethylmalonyl-CoA pathway roughly tripled but never became the major pathway for acetyl-CoA assimilation compared to pyruvate synthase and the reverse TCA cycle. Interestingly, CO_2_ assimilation through RubisCO and the CBB cycle also increased modestly.

Consistent with its role as an anaplerotic pathway, the ethylmalonyl-CoA pathway appears to function only to provide substrates for the TCA cycle in relatively reductive conditions. In these conditions, acetyl-CoA cannot enter the TCA cycle in the oxidative direction because pyruvate formation formate is much more favorable than citrate formation. Consequently, some of the acetyl-CoA is converted to malate and succinate so that 2-oxoglutarate and oxaloacetate can be produced as precursors for amino acid synthesis.

**Biomass Oxidation State.** Finally, we compared the experimentally observed oxidation state of the biomass to the predicted oxidation of biomass from the simulation model. To do so, a measured elemental composition of biomass, such as the average value of C_4_H_7.16_O_2.00_N_0.80_ from Table 1, is compared to the predicted elemental composition. The predicted elemental composition of the biomass is derived from the overall reaction stoichiometry for model growth from the simulations in which the macromolecule ratios of DNA:RNA:protein:lipid produced are fixed in the ratio 1.0: 2.9: 44.1: 8.5, based on the original work of McCulley *et al*. [19]. The predicted elemental composition is not simply that of the elemental composition of the DNA:RNA:protein:lipid ratio because other aspects of metabolism influence the total elemental composition.

To demonstrate how the predicted biomass elemental composition is derived in the model, consider the overall equation for growth on malate under oxidative conditions corresponding to a thermodynamic odds of NADP+:NADPH of 10^10^. The overall stoichiometry is,


$$10C_{4}H_{6}O_{5}+3.8\;{\text{NH}}_{3}+19.5\;\text{NADPH}+2.5\text{​}\;\text{THF}+1.5\;5,10-\text{MTHF}\;+0.05\;5-\text{MTHF}+0.07\;{\text{SO}}_{4}+2.2\;\text{ABP}+21.4\;P_{i}\;\;\to 4.1\;N10-\text{fTHF}+3.1\;p_{i,2}+\text{biomass}+19.5\;\text{NADP}++4.5\;{\text{CO}}_{2,}$$

in which 10 moles of malate (10 C_4_H_6_O_5_) and 3.8 moles of NH_3_ from the environment along with 19.5 moles of NADPH, 2.5 moles of tetrahydrofolate (THF), 1.5 moles of 5,10-methylenetetrahydrofolate, 0.05 moles of 5-methyl tetrahydrofolate, 0.07 moles of sulfate (SO_4_), 2.2 moles of adenine-3,5-bisphosphate (ABP) and 21.4 moles of orthophosphate (P_*i*_) are consumed to produce biomass and 4.1 moles of N10-formyltetrahydrofolate (N10-fTHF), 3.1 moles of diphosphate, 19.5 moles of NADP+ and 4.5 moles of CO_2_. To obtain an estimate of the oxidation state of the biomass in the model, this reaction is simplified by removing the internal metabolites except for the redox pair NADPH and NADP+, replacing biomass by a variable elemental composition, C_4_H_*u*_O_2.04_N_*z*_, and adding a variable amount of water to the right-hand side to give,


$$10C_{4}H_{6}O_{5}+3.8\;{\text{NH}}_{3}+19.5\;\text{NADPH}\to xC_{4}H_{u}O_{2.04}N_{z}+yH_{2}O+4.5{\text{CO}}_{2}$$ (32)

The values of *x*,*y*,*z*, and *u* are obtained by balancing carbon, oxygen, nitrogen and hydrogen, respectively [18]. For growth on malate, the C:O value of 4:2.04 was used as it was supported by multiple measurements reported in Table 5. Use of a C:O value of 4:1.52 from *R. palustris* reported by McCulley [19] increased discrepancy with simulation estimates.

The estimated biomass oxidation states for growth on malate under oxidative, neutral, and reductive conditions are shown in Table 6. Whether the redox conditions within the model are deemed to be oxidative, reductive, or neutral is based, respectively, on whether CO_2_ is produced, consumed, or neither produced nor consumed in significant quantities as before for the idealized model (Table 6). The thermodynamic odds relating the NADPH concentrations to the NADP+ concentrations are also shown for each condition. Regardless of whether CO_2_ was produced or consumed, NADPH was consumed under all conditions, likely due to the coupling of redox reactions to ATP hydrolysis during anabolism, as discussed above.

As can be seen for the rows labeled *oxidized* and *neutral*, the estimated biomass elemental composition is consistent with values that have been observed experimentally, shown in Table 5, while the estimated biomass composition under reduced conditions has a high ratio of hydrogen to carbon. These values are further evidence that *in vivo*, the cell environment is relatively oxidizing despite the anaerobic photosynthetic conditions.

For growth on acetate using the same macromolecular constraints but the measured C:O ratio of 4:1.57 (Table 5), the estimated biomass composition ranged from C_4_H_7.37_O_1.57_N_0.71_ to C_4_H_7.82_O_1.57_N_0.65_, as shown in Table 7. While for malate the H:C ratio between theory and experiment were in agreement, the H:C ratio expected by the model for growth on acetate was higher than the measured elemental composition of C_4_H_6.94_O_1.57_N_0.63_ [51]. This indicated that, as one might expect, the growth substrate significantly affects the biomass composition and macromolecular ratios. In this model for growth on acetate, the macromolecular ratios for growth on malate (see methods) were used to constrain the model. Therefore, for accurate thermo-kinetic modeling of metabolism, accurate elemental biomass composition and ratios of macromolecules (DNA:RNA:protein:lipid) are needed to constrain the model.

Consequently, we measured the macromolecular ratios of DNA:RNA:protein:lipid for *R. rubrum* grown on acetate. For comparison, we also measured the macromolecular ratios produced when *R. rubrum* is grown on malate, ethanol/CO_2_, and butyrate/CO_2_. The results are also shown in Table 5. First, the macromolecular ratios measured for growth on malate of 1.00: 2.96: 39.98: 9.40 are very consistent with those reported by McCully, *et al*. at 1.0: 2.9: 44.1: 8.5. For growth on acetate, the measurements confirmed that *R. rubrum* produces a significantly greater proportion of reduced compounds (lipids) when grown on acetate relative to malate. (Lipid has an elemental formula of approximately C_4_H_7.4_O_1.35_.) The high lipid content is in sharp contrast to estimates of the redox state of biomass based on elemental analysis wherein the hydrogen content is not appreciably higher, C_4_H_6.94_O_1.57_N_0.63_, compared to growth on malate. In fact, despite the high lipid production for cells grown on acetate, ethanol, and butyrate, the oxidation state of the biomass carbon as quantified by elemental analysis would suggest the cell environment to be equal or more oxidized than that for malate-grown cells.

The reason for the predicted high hydrogen content of the acetate-grown biomass by the simulation versus measured by elemental analysis is likely two-fold. First, models are inherently incomplete representations of nature, and in this regard, the inclusion of additional reductive pathways, such as the polyhydroxybutyrate pathway and peptidoglycan pathway, may bring the predicted elemental composition in closer agreement with the measured composition. Alternately, the model may be accurate and the discrepancy is due to the sample preparation process for elemental analysis. In preparation for elemental analysis, cells are heavily washed before lyophilization to remove media components so they do not bias the measurement. During sample washing, it is likely that many soluble, reduced hydrocarbons made by the cell are also washed away, likely affecting not only the C:H:O ratio but also the C:N ratio. For growth on acetate, ethanol, and butyrate, it may very well be that the actual H:C ratio is higher, in line with the macromolecular ratios and model predictions.

What is clear is that macromolecule ratios can vary considerably depending on the growth condition and species (ref. [69] and Table 5). To test the hypothesis that the macromolecular ratios impact the redox state of the cell, we ran the model using DNA:RNA:protein:lipid ratios from two studies for different organisms of 1:6.6:17.7:8.5 from *N. crassa* [70] (lipid composition of 8.5 was estimated here) and 1:17.2:35.2:6.7 from *E. coli* [69]. (The phylogenetic distance to *R. rubrum* is not relevant to the hypothesis.) The estimated biomass compositions are shown in Table 8. The results confirm the hypothesis that varying the ratios of DNA:RNA:protein:fatty acid can significantly impact the redox state. In fact, varying the macromolecule ratios can have a larger impact on biomass redox state than varying the NADPH:NADP+ odds, as can be seen in comparing the middle and top rows of Table 7, in which the NADP+:NADPH ratio changes by 3 orders of magnitude, to the results shown in Table 8, in which the DNA:RNA:protein:fatty acid ratio changes from 1.0: 2.9: 44.1: 8.5 to 1: 6.6 : 17.7 : 8.5 (*N. crassa*) and 1: 17.2: 35.2: 6.7(*E. coli*).

**Table 8:** Elemental biomass composition (bold) estimated from overall chemical reactions for growth on acetate under varying levels of DNA:RNA:protein (P):fatty acid (FA). Estimates were obtained from simulations under redox conditions corresponding to the concentrations of NADP+:NADPH being held at the thermodynamic odds (Eq 24) of 10^7^. The rows labeled with DNA:RNA:protein:lipid values are those from the optimization for growth of the metabolic model while those labeled estimate are estimates of the biomass elemental composition using the stoichiometry found in the model, and can be compared to the similar condition shown for acetate growth shown in Table 7 in which the respective levels were 1.0: 2.9: 44.1: 8.5. The grey rows are for those compounds in the overall chemical equation for the metabolic model that are not used in estimating the elemental composition of the biomass. Abbreviations: ABP: adenine-3,5-bisphosphate; THF: tetrahydrofolate; 5,10-MTHF: 5, 10 methylenetetrahydrofolate; N10-fTHF: N10-formyltetrahydrofolate; P_*i*,2_: diphosphate.

DNA:RNA: protein: lipid	Eq (24) Odds	Overall Reaction		
		0.54 THF + 0.03 5,10-MTHF + 0.03 5-MTHF +		
*N. crassa*	10^7^	0.03 SO_4_ + 0.56 ABP + 22.49 P_*i*_ +	→	0.60 N10-fTHF + 10.56 P_*i*,2_ +
1: 6.6: 17.7: 8.5		10 C_2_H_4_O_2_ + 3.09 NH_3_ + 16.23 NADPH + 3.29 CO_2_		biomass + 16.23 NADP+
esitmate		10 C_2_H_4_O_2_ + 3.09 NH_3_ + 16.23 H_2_ + 3.29 CO_2_	→	5.82 **C**_**4**_**H**_**8.98**_**O**_**2.04**_**N**_**0.53**_ + 14.70 H_2_O
*E. coli*	10^7^	0.94 THF + 0.18 5,10-MTHF + 0.04 5-MTHF + 0.05 SO_4_ + 1.01 ABP + 22.62 P_*i*_ +	→	1.16 N10-fTHF + 10.70 P_*i*,2_ +
1: 17.2: 35.2: 6.7		10 C_2_H_4_O_2_ + 3.77 NH_3_ + 15.35 NADPH + 4.22 CO_2_		biomass + 15.35 NADP+
esitmate		10 C_2_H_4_O_2_ + 3.77 NH_3_ + 15.35 H_2_ + 4.22 CO_2_	→	10 **C**_**4**_**H**_**7.95**_**O**_**2.04**_**N**_**0.62**_ + 16.10 H_2_O

## Discussion

The question we have sought to address is how the balance between oxidation and reduction is maintained during growth on malate and acetate, and in particular, how high potential electrons or reducing equivalents such as NAD(P)H are dissipated. It is clear from the simulation results shown in Fig 3 and the macromolecular mass ratios shown in Table 5 that the redox state of the cell, or ’redox poise’, can drive large changes in macromolecular synthetic pathways. Reducing equivalents appear to be dissipated by modulating the production of reduced carbon compounds such as lipids.

Yet, in comparing the simulation output with experimental results from the literature, it would appear that despite anaerobic phototrophic growth, the cellular environment is not very reductive. The experimental fluxes from metabolic control analysis (MCA) are consistent with NADP+/NADPH odds $\geq 10^{7}$. In fact, the results, even at the high end of the tested range, an odds of 10^10^, are consistent with the experimental data.

These odds correspond to concentration ratios of NADP+:NADPH of approximately 7:7,000, respectively, while the respective ratios for NAD+:NADH concentrations are relatively consistent, in the range from 1,000:1 to 10,000:1 for growth on both malate and acetate (Table 4). Although typical experimentally measured values of these ratios vary roughly from 10:1 to 100:1, the predicted concentration ratios cannot be directly compared to experimental measurements of concentrations because experimental assays measure whole-cell concentrations, including both enzyme-bound and unbound concentrations, while the relevant thermodynamic values in the simulation correspond to only the unbound species. It is the unbound concentrations that control the thermodynamics.

That the redox poise is not more reductive during heterophotosynthetic growth is rather surprising in that photosynthesis drives the production of high potential electrons. However, the degree to which these high potential electrons are available to alter the redox poise of the cell depends on the mode of operation of the electron transport chain. The electron transport chain acts reductively through the redox pair quinone (Q) and quinol (QH_2_). Quinones are a chemical class that includes specific chemical species such as ubiquinone, metaquinone, rhodoquinone, and others that undergo oxidation-reduction between quinone (Q), semi-quinone (${\text{Q}}^{.-}$) and quinol (QH_2_) forms. During phototrophic growth, the ratio of Q/QH_2_ is largely determined by the rate of light-harvesting reactions occurring in the photosynthetic apparatus, which consists of the light-harvesting complex (LHC) and the reaction center (RC). The light reaction has an overall stoichiometry in which two photons (hν) and a quinone oxidize two ferrocytochromes (cyt-c^2 + ^) to produce ferricytochrome (cyt-c^3 + ^) and a quinol using two protons from the cytoplasm ($H_{c}^{+}$),

$$\text{2 h}\nu +{\text{2 cyt-c}}^{2+}+\text{Q}+2{\text{H}}_{\text{c}}^{+}\to {\text{2 cyt-c}}^{3+}+{\text{QH}}_{2}.$$ (29)

Ferricytochrome is recycled back to ferrocytochrome in the cytochrome bc_1_ complex by a multi-step process involving the extraction of two additional protons from the cytoplasm ($H_{c}^{+}$) and the net depositing of four protons to the periplasm ($H_{p}^{+}$) with an overall stoichiometry,

$${\text{2 cyt-c}}^{3+}+{\text{QH}}_{2}+{\text{2 H}}_{\text{c}}^{+}\to {\text{2 cyt-c}}^{2+}+\text{Q}+4{\text{H}}_{\text{p}}^{+}.$$ (30)

The overall process forms a cycle (Cycle 1) involving both the cytochromes and the quinone/quinol pair,


$$\text{Q}+2\;{\text{cyt-c}}^{2+}\;⇌\;{\text{QH}}_{2}+2\;{\text{cyt-c}}^{3+}\;\;↕\;↕\;\text{Q}+2\;{\text{cyt-c}}^{2+}+4{\text{H}}_{\text{p}}^{+}⇌{\text{QH}}_{2}+2\;{\text{cyt-c}}^{3+}2+{\text{H}}_{\text{c}}^{+}\;\;\;\text{Cycle}\;1$$

in which the cycle extracts four protons from the cytoplasm and deposits four protons into the periplasm, thus creating a proton motive force that can be utilized later to drive ATP synthesis via ATP synthase.

Separately, the quinone/quinol pair can also be involved in oxidation-reduction cycles of cytoplasmic NAD(P)+/NAD(P)H and succinate/fumarate (Cycle 2),

$${\text{QH}}_{2}+\text{NAD(P)+}⇌\text{Q + NAD(P)H}$$ (31)

↕↕

$${\text{QH}}_{2}+\text{fumarate}⇌\text{Q + succinate}$$ (32)

Cycle2

The reaction of Eq (31) is generally unfavorable but can be aided by coupling to the proton motive force generated by the cytochrome bc_1_ reaction in Cycle 1 [10], shown in Eq (30). If Cycle 1 and Cycle 2 are strongly coupled either by using the same quinone pool or through the proton motive force, the process of reverse electron flow can produce high levels of the reductant NAD(P)H due to the photosynthetic activity. Although this process would aid the production of NAD(P)H, it may do so at the expense of ATP production by depleting the availability of reduced quinones in Cycle I. In principle, given the abundant energy from photosynthesis, each of Cycles I and II could operate and aid the production of both NAD(P)H and ATP. However, doing so would require sufficient electrons to maintain a high level of reduced quinones to accomplish this. For purple nonsulfur bacteria, the electrons captured in the reduced quinones ultimately originated from a relatively limited pool of donors (e.g., H_2_, sulfide, and organic substrates) compared to the relatively unlimited supply of water in the case of oxygenic phototrophs. A comparison of the reaction fluxes from the simulation model with inferences of reaction fluxes from isotope labeling data suggests that the internal environment remains relatively oxidized. Specifically, during growth on malate, if flux proceeds from malate to oxaloacetate and then to phosphoenolpyruvate via the PEP carboxykinase reaction as indicated by isotope labeling, according to the simulation results, the internal environment of the cell has to be fairly oxidative for this flux to be feasible.

Thus, rather than photosynthesis inducing high levels of NADH or NADPH, the proton gradient across the periplasmic membrane appears to be used mainly for ATP production. Yet, because of the driving force provided to anabolic reactions by ATP, reduction still proceeds despite a relatively oxidative environment (high NAD(P)+ values relative to NAD(P)H). Consequently, in evaluating how cells operate as dissipative structures regarding dissipation of reducing equivalents (Fig 1), it is important to consider the overall thermodynamics rather than just the redox poise of the cell. This is an important issue to consider when engineering *R. rubrum* to overproduce reduced compounds such as ethylene [23].

In addition to adjusting fluxes of redox reactions in central metabolism [19] and using alternate assimilation pathways such as the ethylmalonyl-CoA pathway [20], the simulations suggest that a major route of maintaining redox balance is likely through dissipation of reducing equivalents by increased production of reduced metabolites such as fatty acids. However, this need not be limited to fatty acids.

Varying the relative levels of DNA, RNA, protein, and lipids can impact the redox state of the cell. In support of this hypothesis is the fact that the average oxidation state of carbons in lipids, PHB, and amino acids are much more reduced than the average oxidation states of carbon in RNA and DNA, as shown in Table 9 (see also Table B in S1 Text). For example, the approximate reducing power to convert RNA to amino acids is given by the redox equation,

**Table 9:** Redox states of amino acids and nucleotides. Charge states of atoms were calculated with the Python module OxidationNumberCalculator (https://github.com/Hiwen-STEM/OxidationNumberCalculator). The formula for Nucleotides, RNA, and DNA is for the average 4-mer A(T/U)GC.

Compound	Formula	C	H	O	N	S
**Avg Nucleotide**	C_19_H_20_O_4_N_15_	1.74	1	-2	-3	
**RNA**	C_38_H_42_O_20_N_15_	1.13	1	-2	-3	
**DNA**	C_39_H_44_O_16_N_15_	0.85	1	-2	-3	
**Avg AA** [71]	C_5_H_7.7_O_1.5_N_1.4_S_0.04_	0.05	1	-2	-3	-0.2
**PHB**	C_4_H_6_O_2_	-0.50	1	-2		
**Lipid**	C_4_H_7.4_O_1.35_	-1.18	1	-2		


$${\text{5 C}}_{38}{\text{H}}_{42}{\text{O}}_{16}{\text{N}}_{15}+98.9\text{NADPH}\;\to 38\;{\text{C}}_{5}{\text{H}}_{7.8}{\text{O}}_{1.5}{\text{N}}_{1.4}+32.0{\text{H}}_{2}\text{O}+21.8{\text{NH}}_{3}+98.9\text{NADP+}.$$

If we view, for example, nucleic acids and amino acids as an oxidation-reduction pair analogous to NAD+ and NADH, then we can rationalize that much of the reductant or high potential electrons that would normally require an electron acceptor, such as CO_2_, can likewise be accepted by nucleic acids and converted to amino acids, PHB or lipids. From this perspective, the relatively high protein mass fraction in the DNA:RNA:protein mass ratios of 1.0:2.9:44.1 found in the study by McCulley [19], *et al*. can be understood.

Furthermore, since DNA is produced only during part of the cell cycle, it is apparent that the thermodynamics and redox state of a cell would also have an impact on the dynamics of the cell cycle, and likewise, the cell cycle would have an impact on the redox state of the cell. It is likely that the redox state of the cell undergoes cycling in sync with the *R. rubrum* cell cycle as seen in other cells [72–74]. Such cycling may demonstrate how biological cells, as dissipative structures, use dissipation to drive memory mechanisms [75,76], the DNA processes and dynamics that allow the cell to predict future events from past experience and record memories of new environmental patterns.

Finally, the goal of using a more detailed physics-based, mass action model is to increase the predictive ability of the model and relieve assumptions made in other modeling approaches. For instance, constraint-based flux-modeling does not provide any information on metabolite concentrations or thermodynamics, and the Michaelis-Menten approximation does not represent the thermodynamics correctly. Traditionally, mass action models that included thermodynamics were immensely difficult to implement because of the need for rate parameters. However, it has been shown that the most likely rate parameters can be easily and quickly determined using the method of Lagrangian multipliers [25]. While variability in rate parameters can modulate reaction rates somewhat, they cannot change the direction of the reactions at steady state from those obtained using the most likely parameters. These models then should provide very reasonable and accurate qualitative results.

However, it must be still kept in mind that metabolism is highly complex and models are approximations rather than reality. The two factors that most likely impact the accuracy of the model the most are (1) whether the model is indeed complete enough to capture all the relevant dynamics, and (2) the inferred regulation. That is, even if otherwise highly accurate, the PCO method for inferring regulation assumes that the growth conditions are the same growth conditions as what the actual species in the environment was selected for over generations. While the growth conditions in the model may accurately reflect the laboratory growth conditions, the laboratory conditions and the natural environment usually differ. Thus, the inferred regulation from the PCO method may not reflect what is actually present in the species. Unfortunately, direct and unambiguous experimental assays of enzyme activities are not generally available that would allow for comparisons [77,78].

## Supporting information

S1 Text Supplementary material (PDF)

S1 Table Comparison to MFA model (Excel)

S2 Table Acetate model. (Excel)

S3 Table Malate model. (Excel)
