# Supplementary material for: Redox poise in R. rubrum phototrophic growth drives large-scale changes in macromolecular pathways
Source: PLoS Comput Biol. 2025 Jun 10;21(6):e1013015. doi: 10.1371/journal.pcbi.1013015 (PMC12151479; doi:10.1371/journal.pcbi.1013015)
Supplement: S1 Text — (PDF) [file pcbi.1013015.s001.pdf]

Supplementary Material

Models and Code

The metabolic model was implemented as described in *Methods* and is available in JSON format and executable in a python-based Jupyter notebook at https://zenodo.org/records/10475680.

Fixed Metabolites

Fixed Metabolite	Concentration
(4S)-4,5-DIHYDROXYPENTAN-2,3-DIONE:CYTOPLASM	2.00×10^{-09}
5-METHYLTETRAHYDROFOLATE:CYTOPLASM	2.00×10^{-03}
5,10-METHYLENETETRAHYDROFOLATE:CYTOPLASM	2.00×10^{-03}
A 7,8-DIHYDROFOLATE:CYTOPLASM	2.00×10^{-03}
ACYL-CARRIER PROTEIN:CYTOPLASM	1.00×10^{-04}
ADENOSINE-3',5'-BISPHOSPHATE:CYTOPLASM	1.00×10^{-03}
AN OXIDIZED C-TYPE CYTOCHROME:EXTERNAL	5.85×10^{-26}
AN_OXIDIZED_FERREDOXIN:CYTOPLASM	2.00×10^{-03}
AN_OXIDIZED_THIOREDOXIN:CYTOPLASM	1.00×10^{-04}
Cell Monomer (Eqn 19)	1.00×10^{-09}
CO2:CYTOPLASM	1.00×10^{-04}
COA:CYTOPLASM	1.40×10^{-06}
DIPHOSPHATE:CYTOPLASM	1.00×10^{-04}
FADH2:CYTOPLASM	2.00×10^{-03}
H2O:CYTOPLASM	$5.55 \times 10^{+01}$
N10-FORMYLTETRAHYDROFOLATE:CYTOPLASM	2.00×10^{-03}
NAD+:CYTOPLASM	2.10×10^{-04}
NADP+:CYTOPLASM	8.86×10^{-06}
NADPH:CYTOPLASM	1.20×10^{-06}
NH3:CYTOPLASM	1.00×10^{-04}
ORTHOPHOSPHATE:CYTOPLASM	2.00×10^{-02}
PHOTON:CYTOPLASM	1.00×10^{-04}
SULFATE:CYTOPLASM	1.00×10^{-04}
TETRAHYDROFOLATE:CYTOPLASM	2.00×10^{-03}
UBIQUINOL:CYTOPLASM	2.00×10^{-03}

Table A. The metabolites listed were held fixed as Dirichlet boundary conditions during all steady state optimizations. In addition, either malate or acetate, depending on the growth conditions was also held fixed at a concentration of 10 mM.

Calculation of Reference Free Energies of Reaction

Reference free energies of reaction are taken to be the standard free energies of reaction adjusted for an aqueous solution of ionic strength of 0.15 M and a pH of 7.0. When standard free energy of reaction values cannot be found in eQuilibrator, the reaction free energies were estimated by various schemes listed below.

- Reference free energies for all reactions involving an exchange of indistinguishable metabolites between compartments were assumed to zero.

- Values for the chemical potential for S-adenosyl-L-methionine (SAM) could not be calculated. Consequently, the reference free energies of reaction for the two reactions involving SAM were taken to be zero:
  - S-ADENMETSYN-RXN:

    $$\text{ATP + L-methionine + H2O} \longrightarrow \text{SAM + phosphate + diphosphate}$$

  - RXN-7605:

    $$\text{SAM + tetrahydrofolate} \longrightarrow S\text{-adenosyl-L-homocysteine} \\ + \text{5-methyltetrahydrofolate}$$

- Reference free energies could not be calculated for,

  $$\begin{aligned} \text{RXN-15121: } \quad \text{2-aminobut-2-enoate} &\longrightarrow \text{2-iminobutanoate} \\ \text{RXN-15123 : 2-iminobutanoate + H2O} &\longrightarrow \text{2-oxobutanoate + NH3} \end{aligned}$$

  However, the combined reaction,

  $$\text{2-aminobut-2-enoate + H2O} \longrightarrow \text{2-oxobutanoate + NH3}$$

  had a reference free energy of -34 kJ/mol. Consequently, each reaction was set to have a reference free energy of half the value (-17 kJ/mol) of the combined reaction.

- Reactions that include cytochromes,
  RXN1ZKB-9:

  $$\text{2 photons + quinone + cyt-c(2+)} \quad \longrightarrow \quad \text{quinol + 2 cyt-c(3+)}$$

  1.10.2.2-RXN:

  $$\text{quinol + 2 cyt-c(3+)} \longrightarrow \text{quinone + cyt-c(2+) 4 H+(periplasm)}$$

  were calculated according to the following electrochemical scheme,

2 Cyt-c(2+)	$\longrightarrow$	2 Cyt-c(3+) + 2e-	2* E°	= 0.293V
Ubiquinone + 2 e-	$\longrightarrow$	Ubiquinol	2 * E°	= -0.083 V
2 Cyt-c(2+) + Ubiquinone	$\longrightarrow$	2 Cyt-c(3+) + Ubiquinol	ΔG°	= -z$F\Delta$ E0
				= -40.52 kJ/mol

  such that reactions RXN1ZKB-9 and 1.10.2.2-RXN had reference free energies of -40.52 and +40.52, respectively.

Compound	Formula	C	H	O	N	**S**
ALA	C3H7NO2	0.00	1	-2	-3	
ARG	C6H14N4O2	0.33	1	-2	-3	
ASN	C4H8N2O3	1.00	1	-2	-3	
ASP	C4H7NO4	1.00	1	-2	-3	
CYS	C3H7NO2S	0.67	1	-2	-3	-2
GLN	C5H10N2O3	0.40	1	-2	-3	
GLU	C5H9NO4	0.40	1	-2	-3	
GLY	C2H5NO2	1.00	1	-2	-3	
HIS	C6H9N3O2	0.67	1	-2	-3	
ILE	C6H13NO2	-1.00	1	-2	-3	
LEU	C6H13NO2	-1.00	1	-2	-3	
LYS	C6H14N2O2	-0.67	1	-2	-3	
MET	C5H11NO2S	-0.40	1	-2	-3	-2
PHE	C9H11NO2	-0.44	1	-2	-3	
PRO	C5H9NO2	-0.40	1	-2	-3	
SER	C3H7NO3	0.67	1	-2	-3	
THR	C4H9NO3	0.00	1	-2	-3	
TRP	C11H12N2O2	-0.18	1	-2	-3	
TYR	C9H11NO3	-0.22	1	-2	-3	
VAL	C5H11NO2	-0.80	1	-2	-3	
**Avg AA**	$C_5H_{7.7}O_{1.5}N_{1.4}S_{0.04}$ [71]	0.05	1	-2	-3	-0.2
ADENINE	C5H5N5	2.00	1		-3	
GUANINE	C5H5N5O	2.40	1	-2	-3	
THYMINE	C5H6N2O2	0.80	1	-2	-3	
CYTOSINE	C4H4N3O	1.75	1	-2	-3	
**Avg Nucleotide**	$C_{19}H_{20}O_4N_{15}$	1.74	1	-2	-3	
**DNA**	$C_{39}H_{44}O_{16}N_{15}$	0.85	1	-2	-3	
**RNA**	$C_{38}H_{42}O_{20}N_{15}$	1.13	1	-2	-3	
**Lipid**	$C_4H_{7.4}O_{1.35}$	-1.18	1	-2		
**PHB**	$C_4H_6O_2$	-0.50	1	-2		

Table B. Redox states of amino acids and nucleotides. Charge states of atoms were calculated with the python module OxidationNumberCalculator (https://github.com/Hiwen-STEM/OxidationNumberCalculator).
